# Crystal structure of silver carbonate iodide Ag_10_(CO_3_)_3_I_4_

**DOI:** 10.1107/S2056989021006022

**Published:** 2021-06-25

**Authors:** Ryoji Suzuki, Yuta Watanabe, Hisanori Yamane, Mamoru Kitaura, Kento Uchida, Yuta Matsushima

**Affiliations:** a Chemistry and Chemical Engineering, Yamagata University, 4-3-16 Jonan, Yonezawa, 992-8510, Japan; b Institute of Multidisciplinary Research for Advanced Materials, Tohoku, University, 2-1-1 Katahira, Aoba-ku, Sendai, 980- 8577, Japan; c Faculty of Science, Yamagata University, 1-4-12 Kojirakawa-machi, Yamagata, 990-8560, Japan

**Keywords:** crystal structure, silver carbonate iodide, layered structure

## Abstract

The title silver carbonate iodide, Ag_10_(CO_3_)_3_I_4_, deca­silver(I) tris­(carbonate) tetra­iodide, was recently reported as a precursor of the new superionic conductor Ag_17_(CO_3_)_3_I_11_. Ag_10_(CO_3_)_3_I_4_, was prepared by heating a stoichiometric powder mixture of AgI and Ag_2_CO_3_ at 430 K. A single-crystal suitable for X-ray diffraction analysis was obtained by slow cooling of a melt with an AgI-rich composition down from 453 K. Ag_10_(CO_3_)_3_I_4_ exhibits a layered crystal structure packed along [10], in which Ag atoms are inter­calated between the layers of hexa­gonally close-packed I atoms, and CO_3_ groups. Up to now, Cs_3_Pb_2_(CO_3_)_3_I is the only other compound containing carbonate groups and iodide ions registered in the Inorganic Crystal Structure Database.

## Abstract 2

The new silver carbonate iodide, Ag_10_(CO_3_)_3_I_4_, comprises layers of I atoms, Ag atoms, and CO_3_ groups stacked along [10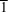].

## Chemical context   

*α*-AgI is a representative superionic conductor of silver ions (Tubandt & Lorenz, 1914 ▸; Boyce & Huberman, 1979 ▸), which is synthesized at 420 K from wurzite-type *β*-AgI and is stable above the phase transition temperature. Many ternary and quaternary compounds containing Ag and I, such as *β*-Ag_3_SI (Takahashi & Yamamoto, 1966 ▸), Ag_4_RbI_5_ (Owens & Argue, 1967 ▸,1970 ▸; Geller, 1967 ▸; Bradley & Greene, 1967*a* ▸,*b* ▸), Ag_4_KI_5_ (Owens & Argue, 1967 ▸; Bradley & Greene, 1966 ▸,1967*b* ▸), Ag_3_KI_4_ (Takahashi *et al.*, 1970 ▸), Ag_19_I_15_P_2_O_7_, Ag_7_I_4_PO_4_ (Takahashi *et al.*, 1972 ▸), Ag_6_I_4_WO_4_ (Takahashi *et al.*, 1973 ▸), and Ag_7_(AsO_4_)I_4_ and Ag_7_I_4_VO_4_ (Scrosati *et al.*, 1975 ▸) have been synthesized and investigated as silver ion conductors. The crystal structures and Ag^+^ ionic conductivity of several silver compounds prepared by combining AgI and a silver oxyanion salt have also been reported (see *Database survey*). However, our recent report on the new superionic conductor Ag_17_(CO_3_)_3_I_11_ (Watanabe *et al.*, 2021 ▸) was the first example of a compound in the AgI–Ag_2_CO_3_ pseudo-binary system. The superionic conductor Ag_17_(CO_3_)_3_I_11_ was prepared by a reaction between AgI and another silver carbonate iodide, *i.e*., Ag_10_(CO_3_)_3_I_4_. Herein, we report the crystal structure analysis of Ag_10_(CO_3_)_3_I_4_ by single-crystal X-ray diffraction (XRD). It should be noted that Cs_3_Pb_2_(CO_3_)_3_I (Liu *et al.*, 2016 ▸) is the only other compound containing carbonate groups and an iodide ion in its chemical composition that can be currently found in the Inorganic Crystal Structure Database (ICSD; Zagorac *et al.*, 2019 ▸).

A bulk Ag_10_(CO_3_)_3_I_4_ sample showed an ionic conductivity of 4.4×10^−6^ S cm^−1^ at room temperature (RT) in the alternating-current impedance method using evaporated Au electrodes. This value is comparable with that of Ag_13_(AsO_4_)_3_I_4_ (6.4×10^−6^ S m^−1^ at 303 K; Pitzschke *et al.*, 2009*a* ▸) and Ag_4_IPO_4_ (3×10^−6^ S m^−1^ at RT; Oleneva *et al.*, 2008 ▸).

## Structural commentary   

**Figure 1:**
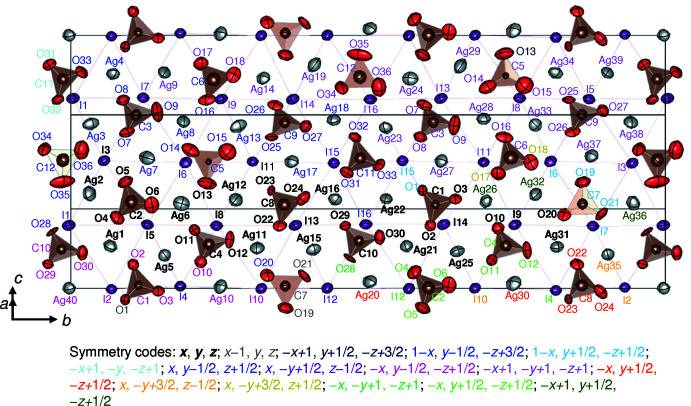
Arrangement of Ag and I atoms and CO_3_ groups in the crystal structure of Ag_10_(CO_3_)_3_I_4_ projected on (10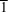). Displacement ellipsoids are drawn at the 99% probability level.

**Figure 2:**
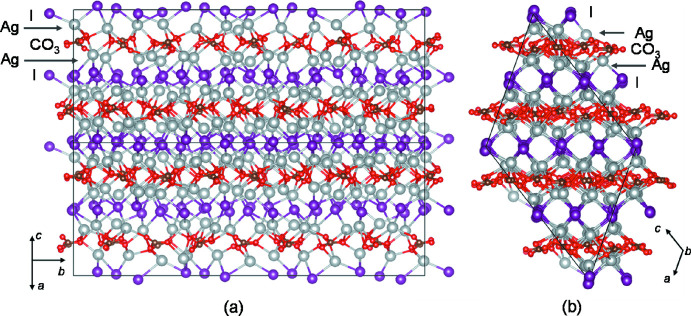
Projections of the crystal structure of Ag_10_(CO_3_)_3_I_4_ along [101] (*a*) and [010] (*b*).

Ag_10_(CO_3_)_3_I_4_ crystallizes in a monoclinic cell with the space group *P*2_1_/*c*. The asymmetric unit of the structure comprises 40 sites for Ag, 16 for I, 12 for C, and 36 for O. The Ag atoms are aligned parallel to (10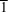), and an Ag layer is sandwiched between a CO_3_ layer and the hexa­gonally aligned I layer, as shown in Fig. 1 ▸. The atomic arrangement of Ag and I atoms and CO_3_ groups in the layers is projected on (10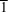) in Fig. 1 ▸, and along [101] and [010] in Fig. 2 ▸ *a* and Fig. 2 ▸ *b*, respectively. Reflecting the complexity of the crystal structure, there is a large variation in the inter­atomic distances. The shortest inter­atomic distance for each atom pair (Ag—O, Ag⋯Ag, Ag—I, *etc*.) is listed in Table 1 ▸.

Fundamental reflections in the XRD pattern were observed at every eight spots along the b* axis in the reciprocal space (Fig. S1 in the supporting information), and a period of *b*/8 = ∼4.43 Å corresponds to the average I⋯I distance in the layer of two-dimensionally closely packed I atoms along the *b* axis. The average I⋯I distance is slightly shorter than that of the close-packed I atom layer of *β*-AgI [4.5910 (11) Å; Yoshiasa *et al.*, 1987 ▸]. The shortest I⋯I length of 3.9564 (11) Å for I10–I12 in Ag_10_(CO_3_)_3_I_4_ lies between the values observed in Ag_13_(AsO_4_)_3_I_4_ [3.9063 (9) Å; Pitzschke *et al.*, 2009*a* ▸] and Ag_26_I_18_(WO_4_)_4_ [4.03 (3) Å; Chan & Geller, 1977 ▸].

The CO_3_ groups are aligned on approximately every *b*/4 period with the oxygen atoms oriented in different directions. The C—O bond lengths are in the range from 1.251 (15) Å for C1—O1 to 1.328 (15) Å for C1—O3, with the average length being 1.290 Å, which is longer than the C—O length in Ag_2_CO_3_ (1.274 Å; Norby *et al.*, 2002 ▸) but close to those in CaCO_3_ (1.282 Å; Maslen *et al.*, 1995 ▸) and other calcite-type carbonates [1.2852 (4) Å in MgCO_3_, 1.2867 (5) Å in MnCO_3_, 1.2869 (5) Å in FeCO_3_, 1.2859 (6) Å in ZnCO_3_, and 1.2853 (4) Å in CaMg(CO_3_)_2_; Effenberger *et al.*, 1981 ▸]. The short O⋯O distance of 2.816 (15) Å for O15⋯O25 belonging to different CO_3_ groups is close to 2.85 Å as observed in MgCO_3_ and ZnCO_3_ (Effenberger *et al.*, 1981 ▸).

The Ag atoms are coordinated by two or three I atoms, and two, three, or four O atoms of the CO_3_ groups, with average distances of 3.20 Å for Ag—I and 2.85 Å for Ag—O. The shortest Ag—I bond length of 2.7140 (14) Å for Ag7—I6 is comparable with that found in Ag_13_(AsO_4_)_3_I_4_ [2.701 (1) Å; Pitzschke *et al.*, 2009*a* ▸] but shorter than the Ag—I bond lengths in *β*-AgI [2.8112 (10) and 2.819 (3) Å; Yoshiasa *et al.*, 1987 ▸]. The shortest Ag—O length is 2.252 (9) Å for Ag12—O23, which is slightly longer than the shortest Ag—O length in Ag_2_CO_3_ (2.245 Å; Norby *et al.*, 2002 ▸).

The shortest Ag⋯Ag distance of 2.9507 (15) Å is observed between Ag17 and Ag27 generated in another Ag layer across the I layer by the symmetry operation *x*, −*y* + 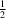, *z* − 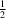. The second shortest Ag⋯Ag distance is 2.9901 (15) Å and corresponds to Ag26⋯Ag32 in the same Ag layer. The shortest Ag⋯Ag distance across the CO_3_ layer is 3.0620 (15) Å for Ag13⋯Ag33. These distances are comparable with the Ag⋯Ag distances reported for Ag_2_CO_3_ [2.8731 (10) Å; Norby *et al.*, 2002 ▸], Ag_8_(CrO_4_)_3_I_2_ [2.8797 (8) Å; Pitzschke *et al.*, 2009*b* ▸], and Ag_3_I(NO_3_)_2_ [2.942 (8) Å; Birnstock & Britton, 1970 ▸]. Such short Ag⋯Ag distances are known to stem from the argentophilic *d* ^10^⋯*d* ^10^ inter­actions between Ag^+^ cations (Schmidbaur & Schier, 2015 ▸; Jansen, 1987 ▸).

## Database survey   

The crystal structures of quaternary inorganic compounds composed of silver and iodide ions and oxyanionic groups, such as Ag_2_I(NO_3_)_2_ (Birnstock & Britton, 1970 ▸), Ag_16_I_12_P_2_O_7_ (Garrett *et al.*, 1982 ▸), Ag_5_IP_2_O_7_ (Adams & Preusser, 1999 ▸), Ag_4_IPO_4_ (Oleneva *et al.*, 2008 ▸), Ag_8_(CrO_4_)_3_I_2_ (Pitzschke *et al.*, 2009*b* ▸), Ag_9_(GeO_4_)_2_I (Pitzschke *et al.*, 2009*c* ▸), Ag_8_I_4_V_2_O_7_ (Adams, 1996 ▸), Ag_13_(AsO_4_)_3_I_4_ (Pitzschke *et al.*, 2009*a* ▸), Ag_4_I_2_SeO_4_ (Pitzschke *et al.*, 2008*a* ▸), Ag_3_ITeO_4_ (Pitzschke *et al.*, 2008*a* ▸), Ag_9_I_3_(IO_3_)_2_(SeO_4_)_2_ (Pitzschke *et al.*, 2008*b* ▸) and Ag_26_I_18_(WO_4_)_4_ (Chan & Geller, 1977 ▸) have been reported.

## Synthesis and crystallization   

As starting materials, powders of AgI and Ag_2_CO_3_ were prepared by precipitation from aqueous solutions containing AgNO_3_ (99.8%, Kanto Chemical) and KI (99.5%, Kanto Chemical) at 323 K, and aqueous solutions of AgNO_3_ and (NH_4_)_2_CO_3_ (>30%_wt_ as NH_3_, Kanto Chemical) at RT, respectively. The obtained polycrystalline solids were thoroughly mixed in an agate mortar at a molar ratio of 4:3 using a small amount of water as a mixing medium.

A bulk brownish sample was prepared at 430 K in a glass tube with one end open. The sample was then powdered with an agate mortar and subjected to powder XRD analysis using a Rigaku MiniFlex 600 powder X-ray diffractometer with Cu Kα radiation (λ = 1.54183 Å) and a 1D detector (Rigaku D/teX Ultra 250).

As for many other silver compounds, Ag_10_(CO_3_)_3_I_4_ is photo-sensitive. In contrast, Ag_10_(CO_3_)_3_I_4_ is thermally stable against heat treatment in air; no substantial mass change was observed up to 473 K in a thermogravimetric and differential thermal analysis (TG–DTA, Rigaku Thermo Evo) (Fig. S2), which was performed under a constant dry air flow of 100 sccm at a ramp rate of 3 K min^−1^, indicating that neither a thermal decomposition nor oxidation occurred. An endothermic effect around 443 K, which can be attributed to the melting of Ag_10_(CO_3_)_3_I_4_, was observed in the TG–DTA analysis. A sample suitable for single-crystal XRD was prepared by subjecting a mixture of the starting powders containing a slight AgI excess composition (AgI:Ag_2_CO_3_ = 4.2:3) to the following heat treatment: after heating to 453 K at a ramp rate of 300 K h^−1^, this temperature was held for 15 min, followed by cooling to 373 K at 1 K h^−1^. The solidified sample was soft and easy to cut with a knife; however, all specimens obtained by cutting showed diffuse XRD spots and were not suitable for single-crystal XRD measurement. Relatively sharp XRD spots were observed from a fragment with a size of 252×245×78 µm, which was spontaneously and accidentally separated by cracking. The surface of the fragment was black and covered with polycrystalline Ag, which was presumably formed by photodegradation during handling. The fragment was fixed on a glass fiber with an ep­oxy resin and mounted on a goniometer under red light through a color filter from an LED lamp, and the XRD data were collected at 90 K with an open-flow nitro­gen gas cooler (Oxford Cobra) in the dark.

## Refinement   

Crystal data, data collection and structure refinement details are summarized in Table 2 ▸. The initial structure model of the space group *P*2_1_/*c* was obtained by intrinsic phasing in the *APEX3* package (Bruker, 2017 ▸). Large positive and negative difference-Fourier peaks were observed, a feature that was probably caused by reflections overlapped with Debye–Scherrer rings from polycrystalline Ag metal on the surface of the crystal (Fig. S1). From the total independent 16240 reflections, 672 reflection data were excluded to lower the difference level within 2.6 e Å^−3^ with *R*1 = 0.0573 for 13157 [*F*_o_ > 4*σ* (*F*_o_)] and 0.0689 for all 15568 data with GooF(*S*) = 1.140. The space groups *P*2_1_, *Pc*, and *P*1 with twin models were also tested; however, neither further effective improvement of the *R* values nor reduction of the residual densities was achieved. The anisotropic displacement parameters of O2, O10, and O15 and the carbon sites were restrained with the ISOR command of *SHELXL* (Sheldrick, 2015*b* ▸) in the final refinement. A Rietveld analysis (Rigaku, 2018 ▸) based on the structural model determined by single-crystal XRD led to monoclinic unit-cell parameters of *a* = 14.2865 (7), *b* = 35.5959 (18), *c* = 17.0182 (8) Å, and *β* = 122.493 (2)° at RT with *R*_F_/*wR* = 0.0294/0.0436 (Fig.  S3), which validates the structural model determined by single-crystal XRD analysis.

## Supplementary Material

Crystal structure: contains datablock(s) global, I. DOI: 10.1107/S2056989021006022/wm5603sup1.cif

Structure factors: contains datablock(s) I. DOI: 10.1107/S2056989021006022/wm5603Isup2.hkl

Rietveld powder data: contains datablock(s) I. DOI: 10.1107/S2056989021006022/wm5603Isup4.rtv

Supplementary figures (Figs. S1-S3). DOI: 10.1107/S2056989021006022/wm5603sup3.pdf

CCDC reference: 2088991

Additional supporting information:  crystallographic information; 3D view; checkCIF report

## Figures and Tables

**Table 1:** Shortest inter­atomic distances (Å) in the crystal structure of Ag_10_(CO_3_)_3_I_4_

Atom pair	Distance
Ag12—O23	2.252 (9)
Ag7—I6	2.7140 (14)
Ag26⋯Ag32 (in-layer)	2.9901 (15)
Ag13⋯Ag33 (out-of-layer; across CO_3_ layer)	3.0620 (15)
Ag17⋯Ag27^i^ (out-of-layer; across I layer)	2.9507 (15)
I10⋯I12^ii^	3.9564 (11)
C1—O1^iii^ (within a CO_3_ group)	1.251 (15)
O25⋯O26 (within a CO_3_ group)	2.206 (14)
O15⋯O25 (between different CO_3_ groups)	2.816 (15)

Symmetry code: (i) *x*, −*y* + {1\over 2}, *z* − {1\over 2}; (ii) −*x*, −*y* + 1, −*z* + 1; (iii) *x* + 1, *y* + {1\over 2}, *z* + {1\over 2}.

**Table 2:** Experimental details

Crystal data	
Chemical formula	Ag_10_(CO_3_)_3_I_4_
*M*_r_	1766.33
Crystal system, space group	Monoclinic, *P*2_1_/*c*
Temperature (K)	90
*a*, *b*, *c* (Å)	14.2342 (11), 35.421 (3), 16.9683 (12)
β (°)	122.725 (3)
*V* (Å^3^)	7197.3 (10)
*Z*	16
Radiation type	Mo *K*α
μ (mm^−1^)	17.53
Crystal size (mm)	0.25 × 0.25 × 0.08

Data collection	
Diffractometer	Bruker D8 goniometer
Absorption correction	Multi-scan (*SADABS*; Krause *et al.*, 2015 ▸)
*T*_min_, *T*_max_	0.376, 0.746
No. of measured, independent and observed [*I* > 2σ(*I*)] reflections	75437, 15568, 13140
*R*_int_	0.043
(sin θ/λ)_max_ (Å^−1^)	0.649

Refinement	
*R*[*F*^2^ > 2σ(*F* ^2^)], *wR*(*F* ^2^), *S*	0.057, 0.126, 1.14
No. of reflections	15568
No. of parameters	937
No. of restraints	90
Δρ_max_, Δρ_min_ (e Å^−3^)	2.22, −2.62

Computer programs: *APEX3* (Bruker, 2017 ▸), *SAINT* (Bruker, 2017 ▸), *SHELXT* (Sheldrick, 2015*a* ▸), *SHELXL* (Sheldrick, 2015*b* ▸), *VESTA* (Momma & Izumi, 2011 ▸), and pubCIF (Westrip, 2010 ▸).

## supplementary crystallographic information

### Crystal data

**Table:**

Ag_10_(CO_3_)_3_I_4_	*F*(000) = 12352
*M**_r_* = 1766.33	*D*_x_ = 6.520 Mg m^−^^3^
Monoclinic, *P*2_1_/*c*	Mo *K*α radiation, λ = 0.71073 Å
*a* = 14.2342 (11) Å	Cell parameters from 4917 reflections
*b* = 35.421 (3) Å	θ = 2.7–35.6°
*c* = 16.9683 (12) Å	µ = 17.53 mm^−^^1^
β = 122.725 (3)°	*T* = 90 K
*V* = 7197.3 (10) Å^3^	Platelet, brown transparent (black agi precipiated on the surface)
*Z* = 16	0.25 × 0.25 × 0.08 mm

### Data collection

**Table:**

Bruker D8 goniometer diffractometer	15568 independent reflections
Radiation source: micro focus sealed tube	13140 reflections with *I* > 2σ(*I*)
Detector resolution: 7.3910 pixels mm^-1^	*R*_int_ = 0.043
ω scans	θ_max_ = 27.5°, θ_min_ = 2.1°
Absorption correction: multi-scan (*SADABS*; Krause *et al.*, 2015)	*h* = −18→18
*T*_min_ = 0.376, *T*_max_ = 0.746	*k* = −45→46
75437 measured reflections	*l* = −22→22

### Refinement

**Table:**

Refinement on *F*^2^	90 restraints
Least-squares matrix: full	Primary atom site location: structure-invariant direct methods
*R*[*F*^2^ > 2σ(*F*^2^)] = 0.057	*w* = 1/[σ^2^(*F*_o_^2^) + (0.0103*P*)^2^ + 615.6616*P*] where *P* = (*F*_o_^2^ + 2*F*_c_^2^)/3
*wR*(*F*^2^) = 0.126	(Δ/σ)_max_ = 0.001
*S* = 1.14	Δρ_max_ = 2.22 e Å^−^^3^
15568 reflections	Δρ_min_ = −2.61 e Å^−^^3^
937 parameters	

### Special details

**Table:**

Geometry. All esds (except the esd in the dihedral angle between two l.s. planes) are estimated using the full covariance matrix. The cell esds are taken into account individually in the estimation of esds in distances, angles and torsion angles; correlations between esds in cell parameters are only used when they are defined by crystal symmetry. An approximate (isotropic) treatment of cell esds is used for estimating esds involving l.s. planes.

### Fractional atomic coordinates and isotropic or equivalent isotropic displacement parameters (Å^2^)

**Table:**

	*x*	*y*	*z*	*U*_iso_*/*U*_eq_	
Ag1	0.08682 (9)	0.06634 (3)	0.20445 (7)	0.0138 (2)	
Ag2	0.27633 (10)	0.03132 (3)	0.41983 (8)	0.0169 (2)	
Ag3	0.55662 (9)	0.46648 (3)	0.19380 (7)	0.0150 (2)	
Ag4	0.26078 (8)	0.57090 (3)	0.60251 (7)	0.01221 (19)	
Ag5	0.03496 (8)	0.15772 (3)	0.17435 (7)	0.0125 (2)	
Ag6	0.23310 (9)	0.18599 (3)	0.38229 (8)	0.0156 (2)	
Ag7	0.43490 (10)	0.37201 (3)	0.05335 (8)	0.0184 (2)	
Ag8	0.57487 (9)	0.31065 (3)	0.19823 (7)	0.0148 (2)	
Ag9	0.25196 (9)	0.84280 (3)	0.09874 (7)	0.0160 (2)	
Ag10	0.07104 (9)	0.75160 (3)	0.45762 (8)	0.0156 (2)	
Ag11	0.07064 (9)	0.31349 (3)	0.19972 (7)	0.0156 (2)	
Ag12	0.24584 (10)	0.27747 (3)	0.39764 (8)	0.0180 (2)	
Ag13	0.55343 (9)	0.21817 (3)	0.18150 (8)	0.0157 (2)	
Ag14	0.27216 (9)	0.67637 (3)	0.12176 (7)	0.0126 (2)	
Ag15	0.05820 (8)	0.40832 (3)	0.19626 (7)	0.0130 (2)	
Ag16	0.24072 (8)	0.44444 (3)	0.40286 (7)	0.0129 (2)	
Ag17	0.42669 (9)	0.12869 (3)	0.03557 (7)	0.0136 (2)	
Ag18	0.58339 (9)	0.05792 (3)	0.20303 (7)	0.0132 (2)	
Ag19	0.22274 (9)	0.59037 (3)	0.06770 (8)	0.0181 (2)	
Ag20	0.04730 (9)	0.00277 (3)	0.43913 (7)	0.0156 (2)	
Ag21	0.08463 (9)	0.56456 (3)	0.20467 (7)	0.0129 (2)	
Ag22	0.24736 (9)	0.53034 (3)	0.40259 (7)	0.0143 (2)	
Ag23	0.46246 (8)	0.47035 (3)	0.32099 (7)	0.0122 (2)	
Ag24	0.27468 (9)	0.42928 (3)	0.12342 (8)	0.0154 (2)	
Ag25	0.04870 (8)	0.65006 (3)	0.19101 (7)	0.0132 (2)	
Ag26	0.76530 (9)	0.19821 (3)	0.10542 (7)	0.0129 (2)	
Ag27	0.57192 (9)	0.37693 (3)	0.46144 (7)	0.0137 (2)	
Ag28	0.41143 (9)	0.30561 (3)	0.29182 (7)	0.0141 (2)	
Ag29	0.22283 (10)	0.34487 (3)	0.07409 (8)	0.0178 (2)	
Ag30	0.05501 (9)	0.24478 (3)	0.44679 (8)	0.0165 (2)	
Ag31	0.08436 (9)	0.81768 (3)	0.20002 (7)	0.0131 (2)	
Ag32	0.73211 (9)	0.28175 (3)	0.08057 (7)	0.0157 (2)	
Ag33	0.45882 (8)	0.21862 (3)	0.30624 (7)	0.0128 (2)	
Ag34	0.26225 (8)	0.18464 (3)	0.09611 (7)	0.0134 (2)	
Ag35	0.03659 (8)	0.59658 (3)	0.67807 (7)	0.0125 (2)	
Ag36	0.77018 (9)	0.44587 (3)	0.11381 (8)	0.0152 (2)	
Ag37	0.54741 (9)	0.12430 (3)	0.44344 (7)	0.0145 (2)	
Ag38	0.42942 (9)	0.05817 (3)	0.30366 (7)	0.0153 (2)	
Ag39	0.24947 (9)	0.09758 (3)	0.10141 (7)	0.0145 (2)	
Ag40	0.07517 (8)	0.49697 (3)	0.46486 (7)	0.0127 (2)	
C1	0.1707 (10)	0.6132 (3)	0.4045 (9)	0.006 (2)	
C2	0.1744 (11)	0.1016 (4)	0.4183 (10)	0.013 (3)	
C3	0.5142 (11)	0.3904 (4)	0.2635 (9)	0.010 (2)	
C4	0.0037 (11)	0.2318 (4)	0.2527 (9)	0.010 (2)	
C5	0.3262 (9)	0.2685 (3)	0.0821 (8)	0.003 (2)	
C6	0.6713 (12)	0.2601 (4)	0.3967 (10)	0.015 (3)	
C7	0.8247 (11)	0.3606 (4)	0.0936 (9)	0.009 (2)	
C8	0.1722 (11)	0.3591 (4)	0.4040 (10)	0.012 (2)	
C9	0.4816 (11)	0.1403 (4)	0.2353 (9)	0.009 (2)	
C10	0.0167 (10)	0.4843 (3)	0.2669 (9)	0.008 (2)	
C11	0.3319 (11)	0.0134 (4)	0.0990 (9)	0.010 (2)	
C12	0.3234 (12)	0.5137 (4)	0.0915 (10)	0.014 (3)	
O1	0.7482 (8)	0.0913 (3)	0.0498 (6)	0.0107 (18)	
O2	0.0858 (8)	0.6049 (3)	0.3234 (7)	0.0104 (18)	
O3	0.1689 (8)	0.6468 (3)	0.4381 (6)	0.0117 (18)	
O4	0.1132 (9)	0.0778 (3)	0.3555 (7)	0.016 (2)	
O5	0.2745 (8)	0.0917 (3)	0.4871 (7)	0.018 (2)	
O6	0.1392 (8)	0.1358 (3)	0.4168 (8)	0.017 (2)	
O7	0.4765 (8)	0.4056 (3)	0.1825 (7)	0.0133 (19)	
O8	0.5459 (8)	0.4123 (3)	0.3353 (7)	0.0127 (19)	
O9	0.5252 (9)	0.3548 (3)	0.2765 (8)	0.019 (2)	
O10	0.0376 (8)	0.7185 (3)	0.3300 (7)	0.0116 (18)	
O11	0.0329 (8)	0.2081 (3)	0.3213 (7)	0.0116 (18)	
O12	0.0199 (8)	0.2671 (3)	0.2713 (7)	0.0129 (19)	
O13	0.2394 (9)	0.2290 (3)	0.4959 (7)	0.021 (2)	
O14	0.3574 (9)	0.2988 (3)	0.1329 (7)	0.020 (2)	
O15	0.3718 (10)	0.2370 (3)	0.1167 (8)	0.024 (2)	
O16	0.5860 (8)	0.2710 (3)	0.3183 (7)	0.015 (2)	
O17	0.2442 (8)	0.7812 (3)	0.0509 (7)	0.016 (2)	
O18	0.3342 (8)	0.7264 (3)	0.0741 (7)	0.0137 (19)	
O19	0.7387 (8)	0.3610 (3)	0.0068 (7)	0.016 (2)	
O20	0.1418 (8)	0.8301 (3)	0.3600 (7)	0.0126 (19)	
O21	0.8723 (8)	0.3927 (3)	0.1296 (7)	0.0128 (19)	
O22	0.0857 (8)	0.3514 (3)	0.3222 (7)	0.0122 (19)	
O23	0.2538 (8)	0.3370 (2)	0.4474 (7)	0.0122 (19)	
O24	0.1684 (8)	0.3910 (3)	0.4422 (7)	0.015 (2)	
O25	0.4524 (8)	0.1625 (3)	0.1640 (6)	0.0125 (19)	
O26	0.5245 (8)	0.1561 (3)	0.3156 (6)	0.0116 (18)	
O27	0.4623 (8)	0.1051 (3)	0.2223 (7)	0.0138 (19)	
O28	0.0239 (8)	0.5311 (3)	0.8153 (6)	0.0119 (19)	
O29	0.0494 (8)	0.4614 (3)	0.3380 (6)	0.0100 (18)	
O30	0.0301 (8)	0.5192 (3)	0.2810 (7)	0.0135 (19)	
O31	0.2471 (8)	0.0360 (3)	0.0549 (7)	0.0116 (18)	
O32	0.4167 (8)	0.0220 (3)	0.1802 (7)	0.0128 (19)	
O33	0.3297 (8)	0.5180 (3)	0.5604 (7)	0.015 (2)	
O34	0.3586 (9)	0.5446 (3)	0.1375 (7)	0.017 (2)	
O35	0.2370 (9)	0.5143 (4)	0.0050 (8)	0.026 (3)	
O36	0.3710 (9)	0.4821 (3)	0.1274 (8)	0.023 (2)	
I1	0.23839 (7)	0.50353 (2)	0.75382 (6)	0.01089 (16)	
I2	0.02321 (7)	0.56825 (2)	0.49450 (6)	0.00922 (16)	
I3	0.51853 (7)	0.05568 (2)	0.49871 (6)	0.01051 (16)	
I4	0.02890 (7)	0.31583 (2)	0.49893 (6)	0.00977 (16)	
I5	0.24870 (7)	0.12856 (2)	0.25332 (6)	0.01133 (17)	
I6	0.48805 (7)	0.30683 (2)	0.00222 (6)	0.01024 (16)	
I7	0.74654 (7)	0.37823 (2)	0.25684 (6)	0.01038 (16)	
I8	0.24565 (7)	0.24507 (2)	0.24870 (6)	0.01053 (16)	
I9	0.23835 (7)	0.74699 (2)	0.25736 (6)	0.01027 (16)	
I10	0.01605 (7)	0.82021 (2)	0.49369 (6)	0.01008 (16)	
I11	0.52303 (7)	0.30574 (2)	0.49127 (6)	0.00945 (16)	
I12	0.00636 (7)	0.06869 (2)	0.49831 (6)	0.01186 (17)	
I13	0.26024 (7)	0.37760 (2)	0.24425 (6)	0.01006 (16)	
I14	0.26343 (7)	0.62687 (2)	0.24731 (6)	0.01021 (16)	
I15	0.47596 (7)	0.05766 (2)	0.00446 (6)	0.00997 (16)	
I16	0.24648 (7)	0.49627 (2)	0.25586 (6)	0.01117 (16)	

### Atomic displacement parameters (Å^2^)

**Table:**

	*U*^11^	*U*^22^	*U*^33^	*U*^12^	*U*^13^	*U*^23^
Ag1	0.0155 (5)	0.0147 (5)	0.0103 (5)	−0.0023 (4)	0.0063 (4)	−0.0016 (4)
Ag2	0.0214 (5)	0.0133 (5)	0.0163 (5)	0.0022 (4)	0.0104 (4)	−0.0014 (4)
Ag3	0.0117 (5)	0.0200 (5)	0.0125 (5)	−0.0045 (4)	0.0060 (4)	−0.0011 (4)
Ag4	0.0085 (4)	0.0131 (5)	0.0127 (5)	0.0004 (3)	0.0041 (4)	−0.0010 (4)
Ag5	0.0108 (4)	0.0120 (5)	0.0137 (5)	0.0006 (3)	0.0060 (4)	−0.0007 (4)
Ag6	0.0137 (5)	0.0197 (5)	0.0153 (5)	−0.0005 (4)	0.0092 (4)	−0.0015 (4)
Ag7	0.0198 (5)	0.0203 (5)	0.0127 (5)	0.0083 (4)	0.0073 (4)	−0.0013 (4)
Ag8	0.0201 (5)	0.0124 (5)	0.0097 (5)	0.0007 (4)	0.0066 (4)	0.0005 (4)
Ag9	0.0224 (5)	0.0119 (5)	0.0095 (5)	0.0014 (4)	0.0060 (4)	−0.0018 (4)
Ag10	0.0169 (5)	0.0145 (5)	0.0146 (5)	0.0022 (4)	0.0080 (4)	−0.0036 (4)
Ag11	0.0204 (5)	0.0155 (5)	0.0095 (5)	0.0008 (4)	0.0073 (4)	0.0005 (4)
Ag12	0.0237 (6)	0.0148 (5)	0.0121 (5)	0.0031 (4)	0.0074 (4)	−0.0013 (4)
Ag13	0.0123 (5)	0.0210 (5)	0.0145 (5)	−0.0065 (4)	0.0076 (4)	−0.0026 (4)
Ag14	0.0134 (5)	0.0141 (5)	0.0109 (5)	−0.0013 (4)	0.0070 (4)	0.0019 (4)
Ag15	0.0089 (4)	0.0159 (5)	0.0133 (5)	0.0027 (4)	0.0053 (4)	0.0013 (4)
Ag16	0.0093 (4)	0.0139 (5)	0.0136 (5)	−0.0010 (4)	0.0050 (4)	0.0005 (4)
Ag17	0.0133 (5)	0.0155 (5)	0.0114 (5)	0.0023 (4)	0.0063 (4)	−0.0016 (4)
Ag18	0.0139 (5)	0.0148 (5)	0.0088 (5)	−0.0009 (4)	0.0048 (4)	−0.0010 (4)
Ag19	0.0187 (5)	0.0147 (5)	0.0185 (5)	0.0036 (4)	0.0085 (4)	0.0055 (4)
Ag20	0.0173 (5)	0.0161 (5)	0.0111 (5)	0.0035 (4)	0.0063 (4)	−0.0024 (4)
Ag21	0.0158 (5)	0.0117 (5)	0.0106 (5)	0.0003 (4)	0.0066 (4)	0.0006 (4)
Ag22	0.0182 (5)	0.0115 (5)	0.0082 (5)	0.0014 (4)	0.0038 (4)	−0.0006 (4)
Ag23	0.0084 (4)	0.0128 (5)	0.0138 (5)	−0.0004 (3)	0.0049 (4)	−0.0011 (4)
Ag24	0.0138 (5)	0.0197 (5)	0.0133 (5)	−0.0020 (4)	0.0077 (4)	0.0005 (4)
Ag25	0.0104 (5)	0.0155 (5)	0.0107 (5)	0.0027 (4)	0.0039 (4)	0.0023 (4)
Ag26	0.0112 (5)	0.0116 (4)	0.0144 (5)	0.0005 (3)	0.0060 (4)	−0.0025 (4)
Ag27	0.0142 (5)	0.0138 (5)	0.0125 (5)	−0.0017 (4)	0.0069 (4)	0.0019 (4)
Ag28	0.0154 (5)	0.0153 (5)	0.0095 (5)	−0.0011 (4)	0.0053 (4)	0.0002 (4)
Ag29	0.0236 (6)	0.0136 (5)	0.0159 (5)	0.0048 (4)	0.0105 (5)	0.0039 (4)
Ag30	0.0190 (5)	0.0178 (5)	0.0148 (5)	0.0010 (4)	0.0106 (4)	−0.0034 (4)
Ag31	0.0131 (5)	0.0140 (5)	0.0091 (5)	−0.0003 (4)	0.0040 (4)	0.0004 (4)
Ag32	0.0208 (5)	0.0124 (5)	0.0113 (5)	−0.0035 (4)	0.0071 (4)	−0.0011 (4)
Ag33	0.0106 (5)	0.0134 (5)	0.0147 (5)	0.0006 (4)	0.0069 (4)	−0.0015 (4)
Ag34	0.0085 (4)	0.0161 (5)	0.0132 (5)	−0.0008 (4)	0.0043 (4)	−0.0014 (4)
Ag35	0.0087 (4)	0.0123 (5)	0.0142 (5)	−0.0008 (3)	0.0047 (4)	0.0003 (4)
Ag36	0.0128 (5)	0.0136 (5)	0.0185 (5)	0.0020 (4)	0.0081 (4)	0.0008 (4)
Ag37	0.0155 (5)	0.0150 (5)	0.0119 (5)	−0.0021 (4)	0.0066 (4)	0.0020 (4)
Ag38	0.0213 (5)	0.0147 (5)	0.0094 (5)	−0.0013 (4)	0.0080 (4)	−0.0011 (4)
Ag39	0.0175 (5)	0.0120 (5)	0.0097 (5)	0.0002 (4)	0.0046 (4)	−0.0025 (4)
Ag40	0.0119 (5)	0.0134 (5)	0.0113 (5)	0.0021 (4)	0.0052 (4)	−0.0010 (4)
C1	0.006 (2)	0.006 (2)	0.006 (2)	0.0001 (10)	0.0032 (14)	−0.0001 (10)
C2	0.013 (3)	0.013 (3)	0.013 (3)	0.0002 (10)	0.0069 (16)	−0.0003 (10)
C3	0.010 (3)	0.010 (3)	0.010 (3)	−0.0003 (10)	0.0053 (15)	0.0001 (10)
C4	0.010 (3)	0.010 (3)	0.010 (3)	0.0001 (10)	0.0054 (15)	0.0003 (10)
C5	0.003 (2)	0.003 (2)	0.003 (2)	0.0000 (10)	0.0019 (14)	0.0001 (10)
C6	0.016 (3)	0.016 (3)	0.015 (3)	0.0000 (10)	0.0085 (17)	−0.0001 (10)
C7	0.009 (2)	0.009 (2)	0.009 (2)	0.0004 (10)	0.0048 (15)	−0.0003 (10)
C8	0.012 (3)	0.012 (3)	0.011 (3)	−0.0003 (10)	0.0063 (16)	0.0000 (10)
C9	0.009 (2)	0.009 (2)	0.009 (2)	0.0002 (10)	0.0048 (15)	−0.0002 (10)
C10	0.008 (2)	0.008 (2)	0.008 (2)	−0.0001 (10)	0.0041 (15)	0.0006 (10)
C11	0.010 (3)	0.010 (3)	0.010 (3)	−0.0002 (10)	0.0054 (15)	−0.0001 (10)
C12	0.014 (3)	0.014 (3)	0.014 (3)	0.0001 (10)	0.0079 (16)	−0.0003 (10)
O1	0.013 (4)	0.007 (4)	0.009 (4)	0.001 (3)	0.004 (4)	0.000 (3)
O2	0.010 (2)	0.010 (2)	0.010 (2)	0.0000 (10)	0.0052 (13)	−0.0005 (10)
O3	0.016 (5)	0.012 (4)	0.006 (4)	0.000 (4)	0.006 (4)	0.000 (3)
O4	0.021 (5)	0.015 (5)	0.013 (5)	−0.001 (4)	0.009 (4)	−0.004 (4)
O5	0.012 (5)	0.018 (5)	0.016 (5)	0.002 (4)	0.002 (4)	0.006 (4)
O6	0.010 (5)	0.016 (5)	0.027 (6)	0.001 (4)	0.011 (4)	−0.001 (4)
O7	0.012 (5)	0.018 (5)	0.008 (5)	−0.003 (4)	0.004 (4)	−0.003 (4)
O8	0.013 (5)	0.014 (5)	0.008 (5)	−0.002 (4)	0.004 (4)	−0.002 (4)
O9	0.025 (6)	0.010 (5)	0.030 (6)	0.003 (4)	0.018 (5)	0.003 (4)
O10	0.012 (2)	0.012 (2)	0.011 (2)	−0.0003 (10)	0.0060 (13)	0.0002 (10)
O11	0.012 (4)	0.009 (4)	0.010 (5)	0.000 (3)	0.004 (4)	−0.001 (3)
O12	0.018 (5)	0.006 (4)	0.021 (5)	0.001 (4)	0.015 (4)	0.002 (4)
O13	0.013 (5)	0.029 (6)	0.014 (5)	0.000 (4)	0.002 (4)	−0.005 (4)
O14	0.020 (5)	0.024 (6)	0.014 (5)	0.004 (4)	0.009 (5)	0.006 (4)
O15	0.023 (3)	0.024 (3)	0.024 (3)	0.0003 (10)	0.0129 (15)	0.0003 (10)
O16	0.012 (5)	0.023 (5)	0.009 (5)	0.001 (4)	0.006 (4)	−0.004 (4)
O17	0.010 (5)	0.016 (5)	0.019 (5)	0.001 (4)	0.005 (4)	0.001 (4)
O18	0.017 (5)	0.016 (5)	0.016 (5)	0.003 (4)	0.014 (4)	0.001 (4)
O19	0.016 (5)	0.019 (5)	0.009 (5)	0.001 (4)	0.004 (4)	−0.004 (4)
O20	0.019 (5)	0.014 (5)	0.009 (5)	0.001 (4)	0.010 (4)	0.001 (4)
O21	0.007 (4)	0.012 (4)	0.016 (5)	−0.005 (3)	0.004 (4)	−0.007 (4)
O22	0.016 (5)	0.014 (5)	0.007 (4)	0.001 (4)	0.007 (4)	0.001 (4)
O23	0.011 (4)	0.005 (4)	0.011 (5)	0.003 (3)	0.000 (4)	−0.001 (3)
O24	0.013 (5)	0.015 (5)	0.017 (5)	0.005 (4)	0.007 (4)	0.005 (4)
O25	0.009 (4)	0.014 (5)	0.006 (4)	−0.004 (3)	−0.001 (4)	0.002 (4)
O26	0.013 (5)	0.018 (5)	0.005 (4)	0.003 (4)	0.006 (4)	0.001 (4)
O27	0.012 (5)	0.011 (4)	0.014 (5)	0.001 (4)	0.004 (4)	−0.003 (4)
O28	0.013 (5)	0.011 (4)	0.006 (4)	−0.003 (3)	0.001 (4)	−0.006 (3)
O29	0.008 (4)	0.010 (4)	0.008 (4)	−0.001 (3)	0.002 (4)	0.002 (3)
O30	0.013 (5)	0.009 (4)	0.019 (5)	−0.002 (3)	0.009 (4)	0.000 (4)
O31	0.011 (4)	0.013 (4)	0.010 (5)	0.002 (3)	0.005 (4)	0.003 (4)
O32	0.009 (4)	0.012 (4)	0.011 (5)	−0.003 (3)	0.001 (4)	0.005 (4)
O33	0.018 (5)	0.012 (5)	0.010 (5)	−0.003 (4)	0.005 (4)	0.006 (4)
O34	0.029 (6)	0.013 (5)	0.008 (5)	−0.004 (4)	0.009 (4)	−0.001 (4)
O35	0.010 (5)	0.048 (7)	0.016 (5)	−0.011 (5)	0.004 (4)	−0.002 (5)
O36	0.022 (6)	0.017 (5)	0.031 (6)	−0.002 (4)	0.015 (5)	−0.001 (5)
I1	0.0093 (4)	0.0134 (4)	0.0081 (4)	−0.0014 (3)	0.0035 (3)	−0.0009 (3)
I2	0.0090 (4)	0.0099 (4)	0.0070 (4)	−0.0008 (3)	0.0032 (3)	0.0001 (3)
I3	0.0110 (4)	0.0106 (4)	0.0087 (4)	0.0012 (3)	0.0045 (3)	0.0018 (3)
I4	0.0097 (4)	0.0107 (4)	0.0075 (4)	−0.0008 (3)	0.0037 (3)	0.0001 (3)
I5	0.0087 (4)	0.0119 (4)	0.0092 (4)	−0.0005 (3)	0.0022 (3)	−0.0018 (3)
I6	0.0119 (4)	0.0114 (4)	0.0086 (4)	−0.0006 (3)	0.0063 (3)	−0.0012 (3)
I7	0.0087 (4)	0.0119 (4)	0.0082 (4)	−0.0006 (3)	0.0029 (3)	0.0009 (3)
I8	0.0087 (4)	0.0124 (4)	0.0079 (4)	0.0002 (3)	0.0028 (3)	−0.0004 (3)
I9	0.0094 (4)	0.0129 (4)	0.0071 (4)	0.0016 (3)	0.0035 (3)	0.0010 (3)
I10	0.0107 (4)	0.0102 (4)	0.0080 (4)	0.0001 (3)	0.0041 (3)	−0.0010 (3)
I11	0.0103 (4)	0.0097 (4)	0.0069 (4)	0.0007 (3)	0.0037 (3)	0.0003 (3)
I12	0.0153 (4)	0.0096 (4)	0.0087 (4)	−0.0010 (3)	0.0052 (3)	−0.0010 (3)
I13	0.0080 (4)	0.0126 (4)	0.0083 (4)	0.0012 (3)	0.0035 (3)	0.0019 (3)
I14	0.0087 (4)	0.0124 (4)	0.0085 (4)	0.0014 (3)	0.0040 (3)	0.0025 (3)
I15	0.0094 (4)	0.0109 (4)	0.0082 (4)	−0.0002 (3)	0.0038 (3)	0.0000 (3)
I16	0.0087 (4)	0.0136 (4)	0.0081 (4)	0.0011 (3)	0.0025 (3)	−0.0001 (3)

### Geometric parameters (Å, º)

**Table:**

Ag1—O4	2.416 (10)	Ag30—I4	2.7587 (14)
Ag1—O30^i^	2.462 (10)	Ag30—O12	2.852 (10)
Ag1—O2^i^	2.621 (9)	Ag30—I10^ix^	2.9063 (14)
Ag1—I2^i^	2.8525 (13)	Ag30—Ag10^ix^	3.0022 (15)
Ag1—I5	2.9644 (13)	Ag30—Ag31^i^	3.3862 (15)
Ag1—I1^ii^	3.0865 (13)	Ag31—O20	2.415 (9)
Ag1—Ag5	3.2978 (15)	Ag31—O12^x^	2.536 (10)
Ag1—Ag2	3.3930 (16)	Ag31—O22^x^	2.538 (10)
Ag2—O35^iii^	2.427 (13)	Ag31—I4^x^	2.8487 (13)
Ag2—O5	2.432 (11)	Ag31—I7^vi^	3.0043 (13)
Ag2—O4	2.561 (10)	Ag31—I9	3.1169 (13)
Ag2—I1^ii^	2.8469 (14)	Ag31—Ag35^viii^	3.0904 (14)
Ag2—I3	3.0712 (14)	Ag31—Ag30^x^	3.3862 (15)
Ag2—Ag36^iv^	3.0846 (15)	Ag32—O18^xi^	2.277 (10)
Ag3—O36	2.310 (11)	Ag32—O20^iv^	2.284 (10)
Ag3—O7	2.398 (10)	Ag32—I9^iv^	2.8293 (14)
Ag3—I1^v^	2.7620 (13)	Ag32—I6	3.1036 (14)
Ag3—O32^vi^	2.777 (9)	Ag32—Ag10^iv^	3.3724 (16)
Ag3—I3^ii^	3.1508 (14)	Ag33—O26	2.376 (10)
Ag3—Ag23	3.0984 (15)	Ag33—O16	2.522 (10)
Ag3—Ag38^vi^	3.2525 (15)	Ag33—O18^iv^	2.532 (10)
Ag3—Ag4^v^	3.2774 (15)	Ag33—I8	2.7989 (13)
Ag4—O33	2.395 (11)	Ag33—O15	2.827 (12)
Ag4—O8^v^	2.430 (10)	Ag33—I6^iii^	3.2480 (14)
Ag4—O1^vi^	2.620 (10)	Ag34—O15	2.323 (11)
Ag4—I2	2.8465 (13)	Ag34—O25	2.429 (10)
Ag4—I7^v^	3.0366 (13)	Ag34—O23^ii^	2.577 (10)
Ag4—Ag9^vii^	3.0588 (15)	Ag34—I4^ii^	2.7982 (13)
Ag4—Ag3^v^	3.2774 (15)	Ag34—I5	3.4141 (14)
Ag5—O10^i^	2.372 (9)	Ag34—Ag39	3.0935 (15)
Ag5—O3^i^	2.495 (10)	Ag35—O29^ix^	2.330 (9)
Ag5—O2^i^	2.556 (9)	Ag35—O22^ix^	2.534 (10)
Ag5—I5	2.7769 (13)	Ag35—O24^ix^	2.536 (10)
Ag5—I4^ii^	3.0768 (14)	Ag35—I7^v^	2.8033 (13)
Ag5—Ag25^i^	3.1071 (15)	Ag35—O21^v^	2.811 (10)
Ag5—Ag6	3.2630 (15)	Ag35—I2	3.1788 (14)
Ag6—O13	2.420 (11)	Ag35—Ag15^ix^	3.0836 (15)
Ag6—O6	2.478 (10)	Ag35—Ag31^vii^	3.0902 (14)
Ag6—O11	2.572 (10)	Ag36—O21	2.303 (10)
Ag6—I6^iii^	3.0645 (14)	Ag36—O35^xi^	2.417 (12)
Ag6—I5	3.0827 (14)	Ag36—O28^v^	2.626 (10)
Ag6—I8	3.1602 (14)	Ag36—I1^v^	2.9272 (14)
Ag6—Ag12	3.2479 (16)	Ag36—I3^ii^	3.0141 (13)
Ag7—O7	2.273 (10)	Ag36—Ag2^vi^	3.0847 (15)
Ag7—O5^ii^	2.313 (10)	Ag36—Ag19^xi^	3.3882 (16)
Ag7—I6	2.7140 (14)	Ag37—O26	2.304 (9)
Ag7—I3^ii^	3.1669 (15)	Ag37—O19^iii^	2.383 (10)
Ag7—Ag37^ii^	3.0480 (16)	Ag37—I3	2.7154 (13)
Ag7—Ag8	3.0725 (15)	Ag37—I6^iii^	2.9276 (14)
Ag8—O9	2.393 (11)	Ag37—Ag7^iii^	3.0479 (16)
Ag8—O16	2.408 (10)	Ag37—Ag38	3.1027 (15)
Ag8—O14	2.698 (11)	Ag38—O27	2.364 (10)
Ag8—I6	2.8571 (14)	Ag38—O32	2.377 (10)
Ag8—I7	3.1721 (13)	Ag38—O34^iv^	2.660 (11)
Ag8—I9^iv^	3.2491 (14)	Ag38—I3	2.8340 (14)
Ag8—Ag9^iv^	3.1717 (15)	Ag38—I1^ii^	3.2245 (14)
Ag8—Ag13	3.2882 (15)	Ag38—I5	3.3447 (14)
Ag9—O17	2.308 (10)	Ag38—Ag3^iv^	3.2525 (15)
Ag9—O3^viii^	2.340 (9)	Ag38—Ag39	3.2837 (15)
Ag9—O9^vi^	2.732 (11)	Ag39—O31	2.312 (9)
Ag9—I7^vi^	2.7431 (14)	Ag39—O24^ii^	2.329 (10)
Ag9—Ag4^viii^	3.0588 (14)	Ag39—O27	2.596 (10)
Ag9—Ag8^vi^	3.1717 (15)	Ag39—I5	2.8069 (14)
Ag9—Ag27^vi^	3.3996 (16)	Ag40—O29	2.346 (9)
Ag10—O10	2.274 (10)	Ag40—O31^iii^	2.376 (10)
Ag10—O17^vii^	2.391 (10)	Ag40—I2	2.7526 (13)
Ag10—I10	2.7225 (13)	Ag40—I2^ix^	2.9693 (13)
Ag10—I4^ix^	3.0668 (14)	Ag40—Ag40^ix^	2.968 (2)
Ag10—Ag30^ix^	3.0022 (16)	Ag40—O30	2.932 (10)
Ag10—Ag11^x^	3.1906 (15)	C1—O1^vi^	1.251 (15)
Ag10—Ag32^vi^	3.3724 (16)	C1—O2	1.282 (15)
Ag11—O12	2.377 (9)	C1—O3	1.328 (15)
Ag11—O22	2.384 (9)	C2—O4	1.264 (17)
Ag11—O20^i^	2.684 (10)	C2—O6	1.305 (17)
Ag11—I10^i^	2.8234 (13)	C2—O5	1.310 (17)
Ag11—I8	3.2455 (14)	C3—O9	1.272 (16)
Ag11—I13	3.2872 (14)	C3—O7	1.293 (16)
Ag11—Ag12	3.1734 (15)	C3—O8	1.302 (16)
Ag11—Ag10^i^	3.1905 (15)	C4—O12	1.279 (16)
Ag11—Ag15	3.3624 (15)	C4—O10^i^	1.283 (16)
Ag12—O23	2.252 (9)	C4—O11	1.307 (16)
Ag12—O13	2.428 (12)	C5—O15	1.267 (16)
Ag12—O12	2.761 (10)	C5—O14	1.295 (16)
Ag12—I8	2.7745 (14)	C5—O13^ii^	1.312 (15)
Ag13—O15	2.294 (12)	C6—O17^iv^	1.281 (17)
Ag13—O25	2.363 (9)	C6—O16	1.283 (17)
Ag13—I9^iv^	2.7529 (13)	C6—O18^iv^	1.310 (17)
Ag13—O16	2.817 (10)	C7—O20^iv^	1.267 (16)
Ag13—I11^ii^	3.1325 (14)	C7—O21	1.297 (15)
Ag13—Ag33	3.0620 (15)	C7—O19	1.311 (16)
Ag13—Ag14^iv^	3.2462 (15)	C8—O23	1.256 (16)
Ag14—O18	2.313 (10)	C8—O22	1.291 (17)
Ag14—O19^xi^	2.484 (10)	C8—O24	1.319 (17)
Ag14—O26^vi^	2.589 (10)	C9—O27	1.272 (16)
Ag14—I14	2.8131 (13)	C9—O26	1.282 (16)
Ag14—I10^viii^	3.0760 (13)	C9—O25	1.306 (16)
Ag14—Ag19	3.1494 (15)	C10—O30	1.256 (15)
Ag14—Ag13^vi^	3.2463 (15)	C10—O28^ix^	1.305 (16)
Ag15—O21^xii^	2.313 (9)	C10—O29	1.312 (15)
Ag15—O28^ix^	2.400 (9)	C11—O33^ii^	1.284 (16)
Ag15—I13	2.7543 (13)	C11—O32	1.284 (16)
Ag15—O22	2.808 (9)	C11—O31	1.297 (16)
Ag15—I12^ii^	3.1320 (14)	C12—O36	1.279 (18)
Ag15—Ag35^ix^	3.0836 (15)	C12—O34	1.280 (17)
Ag15—Ag16	3.2907 (15)	C12—O35	1.310 (18)
Ag16—O29	2.397 (9)	O1—C1^iv^	1.251 (15)
Ag16—O24	2.415 (10)	O1—Ag22^iv^	2.294 (9)
Ag16—O31^iii^	2.625 (10)	O1—Ag27^ii^	2.396 (9)
Ag16—I15^iii^	2.8193 (13)	O2—Ag5^x^	2.555 (9)
Ag16—I16	3.1336 (13)	O3—Ag9^vii^	2.340 (9)
Ag16—Ag22	3.0444 (14)	O3—Ag26^vi^	2.342 (9)
Ag17—O25	2.336 (10)	O3—Ag5^x^	2.495 (10)
Ag17—O23^ii^	2.407 (9)	O4—Ag21^i^	2.467 (11)
Ag17—I15	2.7388 (13)	O5—Ag7^iii^	2.312 (10)
Ag17—I11^ii^	2.9924 (13)	O5—Ag24^iii^	2.429 (11)
Ag17—Ag27^ii^	2.9507 (15)	O6—Ag25^i^	2.338 (10)
Ag18—O34^iv^	2.409 (10)	O6—Ag29^iii^	2.362 (11)
Ag18—O32	2.530 (9)	O8—Ag4^v^	2.430 (10)
Ag18—O27	2.545 (10)	O10—C4^x^	1.283 (16)
Ag18—I15	2.8498 (13)	O10—Ag5^x^	2.373 (9)
Ag18—I16^iv^	3.0485 (13)	O10—Ag26^vi^	2.503 (10)
Ag18—I14^iv^	3.0730 (13)	O11—Ag25^i^	2.314 (9)
Ag18—Ag23^iv^	3.1508 (14)	O12—Ag31^i^	2.536 (9)
Ag19—O34	2.300 (11)	O13—C5^iii^	1.312 (15)
Ag19—O19^xi^	2.370 (11)	O17—C6^vi^	1.281 (17)
Ag19—O21^xi^	2.915 (10)	O17—Ag10^viii^	2.391 (10)
Ag19—I12^x^	2.9205 (15)	O18—C6^vi^	1.310 (17)
Ag19—O35	2.944 (13)	O18—Ag32^xi^	2.277 (10)
Ag19—I14	3.0637 (15)	O18—Ag33^vi^	2.532 (10)
Ag19—Ag36^xi^	3.3883 (16)	O19—Ag19^xi^	2.370 (11)
Ag20—O28^ii^	2.282 (10)	O19—Ag37^ii^	2.383 (10)
Ag20—O35^iii^	2.376 (10)	O19—Ag14^xi^	2.484 (10)
Ag20—I12	2.7264 (13)	O20—C7^vi^	1.267 (16)
Ag20—I12^xiii^	2.9991 (14)	O20—Ag32^vi^	2.284 (10)
Ag20—Ag20^xiii^	3.021 (2)	O21—Ag15^xiv^	2.313 (9)
Ag20—Ag21^i^	3.0523 (15)	O22—Ag35^ix^	2.535 (10)
Ag21—O30	2.440 (10)	O22—Ag31^i^	2.538 (10)
Ag21—O2	2.462 (9)	O23—Ag17^iii^	2.407 (9)
Ag21—O4^x^	2.467 (11)	O23—Ag34^iii^	2.577 (10)
Ag21—I12^x^	2.9562 (13)	O24—Ag39^iii^	2.329 (10)
Ag21—I16	3.1225 (13)	O24—Ag35^ix^	2.536 (10)
Ag21—I14	3.1435 (13)	O26—Ag14^iv^	2.589 (10)
Ag21—Ag20^x^	3.0524 (15)	O28—C10^ix^	1.305 (16)
Ag21—Ag25	3.0592 (14)	O28—Ag20^iii^	2.282 (10)
Ag21—Ag22	3.1148 (14)	O28—Ag15^ix^	2.400 (9)
Ag22—O1^vi^	2.294 (9)	O29—Ag35^ix^	2.330 (9)
Ag22—O33	2.311 (10)	O30—Ag1^x^	2.462 (10)
Ag22—O30	2.661 (10)	O31—Ag40^ii^	2.376 (10)
Ag22—I16	2.7609 (14)	O32—Ag23^iv^	2.520 (10)
Ag22—Ag40	3.3647 (16)	O33—C11^iii^	1.284 (16)
Ag23—O8	2.321 (10)	O33—Ag23^v^	2.556 (10)
Ag23—O32^vi^	2.520 (10)	O34—Ag18^vi^	2.410 (10)
Ag23—O33^v^	2.556 (10)	O35—Ag20^ii^	2.376 (10)
Ag23—I16	2.8004 (13)	O35—Ag36^xi^	2.417 (12)
Ag23—O36	2.835 (12)	O35—Ag2^ii^	2.427 (13)
Ag23—I15^iii^	3.1727 (14)	I1—Ag3^v^	2.7620 (13)
Ag23—Ag18^vi^	3.1507 (14)	I1—Ag2^iii^	2.8470 (14)
Ag23—Ag24	3.2879 (15)	I1—Ag36^v^	2.9272 (14)
Ag24—O36	2.299 (11)	I1—Ag1^iii^	3.0867 (13)
Ag24—O5^ii^	2.429 (11)	I1—Ag38^iii^	3.2246 (14)
Ag24—O7	2.617 (10)	I2—Ag1^x^	2.8525 (13)
Ag24—I13	2.8377 (14)	I2—Ag40^ix^	2.9692 (13)
Ag24—Ag29	3.0848 (15)	I3—Ag36^iii^	3.0141 (13)
Ag24—I12^ii^	3.2144 (14)	I3—Ag3^iii^	3.1509 (14)
Ag25—O11^x^	2.314 (9)	I3—Ag7^iii^	3.1669 (15)
Ag25—O6^x^	2.338 (10)	I4—Ag34^iii^	2.7982 (13)
Ag25—O2	2.569 (9)	I4—Ag31^i^	2.8487 (13)
Ag25—I14	2.7879 (13)	I4—Ag10^ix^	3.0670 (14)
Ag25—I10^viii^	3.2942 (13)	I4—Ag5^iii^	3.0769 (14)
Ag25—Ag5^x^	3.1070 (15)	I6—Ag37^ii^	2.9276 (14)
Ag26—O3^iv^	2.342 (9)	I6—Ag6^ii^	3.0645 (14)
Ag26—O10^iv^	2.503 (10)	I6—Ag33^ii^	3.2478 (14)
Ag26—O17^xi^	2.683 (11)	I7—Ag9^iv^	2.7430 (14)
Ag26—I11^ii^	2.9055 (13)	I7—Ag35^v^	2.8034 (13)
Ag26—I9^iv^	2.9222 (13)	I7—Ag31^iv^	3.0042 (13)
Ag26—Ag32	2.9901 (15)	I7—Ag4^v^	3.0367 (13)
Ag27—O8	2.330 (10)	I9—Ag13^vi^	2.7528 (13)
Ag27—O1^iii^	2.396 (9)	I9—Ag32^vi^	2.8294 (14)
Ag27—I11	2.7347 (13)	I9—Ag26^vi^	2.9223 (13)
Ag27—O9	2.941 (11)	I9—Ag8^vi^	3.2491 (14)
Ag27—I15^iii^	2.9739 (13)	I10—Ag11^x^	2.8234 (13)
Ag27—Ag17^iii^	2.9508 (15)	I10—Ag30^ix^	2.9062 (14)
Ag27—Ag9^iv^	3.3997 (16)	I10—Ag29^x^	3.0691 (15)
Ag28—O14	2.383 (11)	I10—Ag14^vii^	3.0759 (13)
Ag28—O9	2.488 (11)	I11—Ag26^iii^	2.9055 (13)
Ag28—O16	2.584 (10)	I11—Ag17^iii^	2.9925 (13)
Ag28—I11	2.8574 (13)	I11—Ag13^iii^	3.1326 (14)
Ag28—I8	2.9725 (13)	I12—Ag19^i^	2.9205 (15)
Ag28—I13	3.1476 (13)	I12—Ag21^i^	2.9562 (13)
Ag28—Ag33	3.1352 (15)	I12—Ag20^xiii^	2.9990 (14)
Ag29—O14	2.292 (11)	I12—Ag15^iii^	3.1320 (14)
Ag29—O6^ii^	2.362 (11)	I12—Ag24^iii^	3.2144 (14)
Ag29—I13	2.8812 (14)	I14—Ag18^vi^	3.0729 (13)
Ag29—O5^ii^	2.989 (11)	I15—Ag16^ii^	2.8192 (13)
Ag29—I10^i^	3.0690 (15)	I15—Ag27^ii^	2.9741 (13)
Ag29—O13^ii^	3.001 (12)	I15—Ag23^ii^	3.1726 (14)
Ag30—O13	2.352 (11)	I16—Ag18^vi^	3.0486 (13)
Ag30—O11	2.366 (10)		
			
O4—Ag1—O30^i^	74.6 (3)	O20—Ag31—I7^vi^	81.7 (2)
O4—Ag1—I2^i^	157.3 (3)	O12^x^—Ag31—I7^vi^	158.8 (2)
O30^i^—Ag1—I2^i^	99.6 (2)	O22^x^—Ag31—I7^vi^	106.0 (2)
O4—Ag1—I5	85.8 (2)	I4^x^—Ag31—I7^vi^	99.83 (4)
O30^i^—Ag1—I5	160.3 (2)	O20—Ag31—Ag35^viii^	82.8 (2)
I2^i^—Ag1—I5	99.20 (4)	O12^x^—Ag31—Ag35^viii^	127.2 (2)
O4—Ag1—I1^ii^	99.3 (2)	O22^x^—Ag31—Ag35^viii^	52.4 (2)
O30^i^—Ag1—I1^ii^	80.8 (2)	I4^x^—Ag31—Ag35^viii^	86.17 (4)
I2^i^—Ag1—I1^ii^	101.31 (4)	I7^vi^—Ag31—Ag35^viii^	54.75 (3)
I5—Ag1—I1^ii^	101.33 (4)	O20—Ag31—I9	92.4 (2)
O4—Ag1—Ag5	83.8 (2)	O12^x^—Ag31—I9	76.0 (2)
O30^i^—Ag1—Ag5	125.1 (2)	O22^x^—Ag31—I9	151.3 (2)
I2^i^—Ag1—Ag5	81.94 (4)	I4^x^—Ag31—I9	101.74 (4)
I5—Ag1—Ag5	52.32 (3)	I7^vi^—Ag31—I9	99.32 (4)
I1^ii^—Ag1—Ag5	153.39 (4)	Ag35^viii^—Ag31—I9	154.01 (4)
O35^iii^—Ag2—O5	104.0 (4)	O18^xi^—Ag32—O20^iv^	110.0 (3)
O35^iii^—Ag2—O4	103.6 (3)	O18^xi^—Ag32—I9^iv^	144.7 (3)
O5—Ag2—O4	52.9 (3)	O20^iv^—Ag32—I9^iv^	103.2 (2)
O35^iii^—Ag2—I1^ii^	108.6 (3)	O18^xi^—Ag32—Ag26	88.4 (2)
O5—Ag2—I1^ii^	143.3 (2)	O20^iv^—Ag32—Ag26	130.8 (3)
O4—Ag2—I1^ii^	102.4 (2)	I9^iv^—Ag32—Ag26	60.21 (3)
O35^iii^—Ag2—I3	119.6 (3)	O18^xi^—Ag32—I6	82.7 (2)
O5—Ag2—I3	80.9 (2)	O20^iv^—Ag32—I6	113.9 (2)
O4—Ag2—I3	123.4 (2)	I9^iv^—Ag32—I6	94.86 (4)
I1^ii^—Ag2—I3	96.94 (4)	Ag26—Ag32—I6	113.49 (4)
O35^iii^—Ag2—Ag36^iv^	50.3 (3)	O18^xi^—Ag32—Ag10^iv^	66.9 (2)
O5—Ag2—Ag36^iv^	153.5 (3)	O20^iv^—Ag32—Ag10^iv^	77.7 (2)
O4—Ag2—Ag36^iv^	119.9 (2)	I9^iv^—Ag32—Ag10^iv^	110.28 (4)
I1^ii^—Ag2—Ag36^iv^	58.98 (3)	Ag26—Ag32—Ag10^iv^	68.25 (3)
I3—Ag2—Ag36^iv^	115.74 (4)	I6—Ag32—Ag10^iv^	149.57 (4)
O36—Ag3—O7	78.9 (4)	O26—Ag33—O16	116.2 (3)
O36—Ag3—I1^v^	142.7 (3)	O26—Ag33—O18^iv^	78.6 (3)
O7—Ag3—I1^v^	138.2 (2)	O16—Ag33—O18^iv^	51.8 (3)
O36—Ag3—Ag23	61.2 (3)	O26—Ag33—I8	130.4 (2)
O7—Ag3—Ag23	74.3 (2)	O16—Ag33—I8	112.2 (2)
I1^v^—Ag3—Ag23	122.58 (4)	O18^iv^—Ag33—I8	144.6 (2)
O36—Ag3—I3^ii^	92.6 (3)	O26—Ag33—Ag13	74.8 (2)
O7—Ag3—I3^ii^	83.5 (2)	O16—Ag33—Ag13	59.7 (2)
I1^v^—Ag3—I3^ii^	89.18 (4)	O18^iv^—Ag33—Ag13	78.4 (2)
Ag23—Ag3—I3^ii^	148.13 (4)	I8—Ag33—Ag13	124.03 (5)
O36—Ag3—Ag38^vi^	79.2 (3)	O26—Ag33—Ag28	169.0 (2)
O7—Ag3—Ag38^vi^	157.2 (2)	O16—Ag33—Ag28	53.0 (2)
I1^v^—Ag3—Ag38^vi^	64.24 (3)	O18^iv^—Ag33—Ag28	93.6 (2)
Ag23—Ag3—Ag38^vi^	89.60 (4)	I8—Ag33—Ag28	59.80 (3)
I3^ii^—Ag3—Ag38^vi^	103.73 (4)	Ag13—Ag33—Ag28	96.18 (4)
O36—Ag3—Ag4^v^	131.1 (3)	O26—Ag33—I6^iii^	81.0 (2)
O7—Ag3—Ag4^v^	78.0 (2)	O16—Ag33—I6^iii^	115.6 (2)
I1^v^—Ag3—Ag4^v^	73.65 (4)	O18^iv^—Ag33—I6^iii^	76.1 (2)
Ag23—Ag3—Ag4^v^	71.24 (3)	I8—Ag33—I6^iii^	87.98 (4)
I3^ii^—Ag3—Ag4^v^	126.53 (4)	Ag13—Ag33—I6^iii^	147.73 (4)
Ag38^vi^—Ag3—Ag4^v^	112.42 (4)	Ag28—Ag33—I6^iii^	104.82 (4)
O33—Ag4—O8^v^	76.7 (3)	O26—Ag33—Ag34	78.9 (2)
O33—Ag4—I2	108.5 (2)	O16—Ag33—Ag34	120.0 (2)
O8^v^—Ag4—I2	163.8 (2)	O18^iv^—Ag33—Ag34	146.5 (2)
O33—Ag4—I7^v^	153.1 (2)	I8—Ag33—Ag34	67.94 (3)
O8^v^—Ag4—I7^v^	91.4 (2)	Ag13—Ag33—Ag34	71.85 (4)
I2—Ag4—I7^v^	89.55 (4)	Ag28—Ag33—Ag34	104.45 (4)
O33—Ag4—Ag9^vii^	142.6 (2)	I6^iii^—Ag33—Ag34	124.16 (4)
O8^v^—Ag4—Ag9^vii^	77.8 (2)	O15—Ag34—O25	72.6 (4)
I2—Ag4—Ag9^vii^	89.91 (4)	O15—Ag34—O23^ii^	93.3 (4)
I7^v^—Ag4—Ag9^vii^	53.49 (3)	O25—Ag34—O23^ii^	79.6 (3)
O33—Ag4—Ag3^v^	77.3 (2)	O15—Ag34—I4^ii^	125.4 (3)
O8^v^—Ag4—Ag3^v^	64.8 (2)	O25—Ag34—I4^ii^	160.2 (2)
I2—Ag4—Ag3^v^	130.88 (4)	O23^ii^—Ag34—I4^ii^	90.5 (2)
I7^v^—Ag4—Ag3^v^	75.85 (3)	O15—Ag34—Ag39	146.7 (3)
Ag9^vii^—Ag4—Ag3^v^	115.22 (4)	O25—Ag34—Ag39	74.4 (2)
O10^i^—Ag5—O3^i^	79.0 (3)	O23^ii^—Ag34—Ag39	76.1 (2)
O10^i^—Ag5—O2^i^	112.3 (3)	I4^ii^—Ag34—Ag39	86.64 (4)
O3^i^—Ag5—O2^i^	52.0 (3)	O15—Ag34—Ag33	56.4 (3)
O10^i^—Ag5—I5	134.3 (2)	O25—Ag34—Ag33	62.3 (2)
O3^i^—Ag5—I5	146.0 (2)	O23^ii^—Ag34—Ag33	136.0 (2)
O2^i^—Ag5—I5	106.9 (2)	I4^ii^—Ag34—Ag33	132.24 (4)
O10^i^—Ag5—I4^ii^	84.8 (2)	Ag39—Ag34—Ag33	110.85 (4)
O3^i^—Ag5—I4^ii^	83.9 (2)	O29^ix^—Ag35—O22^ix^	108.9 (3)
O2^i^—Ag5—I4^ii^	124.8 (2)	O29^ix^—Ag35—O24^ix^	75.8 (3)
I5—Ag5—I4^ii^	91.75 (4)	O22^ix^—Ag35—O24^ix^	51.8 (3)
O10^i^—Ag5—Ag25^i^	76.6 (2)	O29^ix^—Ag35—I7^v^	136.3 (2)
O3^i^—Ag5—Ag25^i^	78.4 (2)	O22^ix^—Ag35—I7^v^	112.2 (2)
O2^i^—Ag5—Ag25^i^	52.9 (2)	O24^ix^—Ag35—I7^v^	143.6 (2)
I5—Ag5—Ag25^i^	111.60 (4)	O29^ix^—Ag35—Ag15^ix^	68.4 (2)
I4^ii^—Ag5—Ag25^i^	156.42 (4)	O22^ix^—Ag35—Ag15^ix^	59.0 (2)
O10^i^—Ag5—Ag6	82.1 (2)	O24^ix^—Ag35—Ag15^ix^	79.8 (2)
O3^i^—Ag5—Ag6	147.4 (2)	I7^v^—Ag35—Ag15^ix^	123.08 (4)
O2^i^—Ag5—Ag6	113.8 (2)	O29^ix^—Ag35—Ag31^vii^	161.4 (2)
I5—Ag5—Ag6	60.75 (3)	O22^ix^—Ag35—Ag31^vii^	52.5 (2)
I4^ii^—Ag5—Ag6	120.56 (4)	O24^ix^—Ag35—Ag31^vii^	90.5 (2)
Ag25^i^—Ag5—Ag6	71.49 (3)	I7^v^—Ag35—Ag31^vii^	61.06 (3)
O10^i^—Ag5—Ag1	162.4 (2)	Ag15^ix^—Ag35—Ag31^vii^	97.01 (4)
O3^i^—Ag5—Ag1	92.1 (2)	O29^ix^—Ag35—I2	81.8 (2)
O2^i^—Ag5—Ag1	51.3 (2)	O22^ix^—Ag35—I2	123.4 (2)
I5—Ag5—Ag1	57.66 (3)	O24^ix^—Ag35—I2	80.4 (2)
I4^ii^—Ag5—Ag1	109.61 (4)	I7^v^—Ag35—I2	87.50 (4)
Ag25^i^—Ag5—Ag1	86.73 (4)	Ag15^ix^—Ag35—I2	147.46 (4)
Ag6—Ag5—Ag1	98.25 (4)	Ag31^vii^—Ag35—I2	108.73 (4)
O13—Ag6—O6	93.2 (4)	O21—Ag36—O35^xi^	108.3 (4)
O13—Ag6—O11	71.7 (3)	O21—Ag36—I1^v^	134.1 (3)
O6—Ag6—O11	72.0 (3)	O35^xi^—Ag36—I1^v^	106.4 (3)
O13—Ag6—I6^iii^	84.1 (3)	O21—Ag36—I3^ii^	121.0 (2)
O6—Ag6—I6^iii^	119.9 (2)	O35^xi^—Ag36—I3^ii^	88.3 (3)
O11—Ag6—I6^iii^	154.0 (2)	I1^v^—Ag36—I3^ii^	88.88 (4)
O13—Ag6—I5	174.2 (3)	O21—Ag36—Ag2^vi^	151.9 (2)
O6—Ag6—I5	87.1 (2)	O35^xi^—Ag36—Ag2^vi^	50.6 (3)
O11—Ag6—I5	113.8 (2)	I1^v^—Ag36—Ag2^vi^	56.46 (3)
I6^iii^—Ag6—I5	90.70 (4)	I3^ii^—Ag36—Ag2^vi^	80.62 (4)
O13—Ag6—I8	99.3 (3)	O26—Ag37—O19^iii^	82.2 (3)
O6—Ag6—I8	153.1 (3)	O26—Ag37—I3	142.3 (2)
O11—Ag6—I8	89.4 (2)	O19^iii^—Ag37—I3	112.9 (2)
I6^iii^—Ag6—I8	85.16 (3)	O26—Ag37—I6^iii^	89.6 (2)
I5—Ag6—I8	82.76 (3)	O19^iii^—Ag37—I6^iii^	97.7 (3)
O13—Ag6—Ag12	48.0 (3)	I3—Ag37—I6^iii^	120.18 (5)
O6—Ag6—Ag12	135.3 (2)	O26—Ag37—Ag7^iii^	134.5 (2)
O11—Ag6—Ag12	74.4 (2)	O19^iii^—Ag37—Ag7^iii^	124.4 (3)
I6^iii^—Ag6—Ag12	82.49 (4)	I3—Ag37—Ag7^iii^	66.37 (4)
I5—Ag6—Ag12	133.87 (4)	I6^iii^—Ag37—Ag7^iii^	53.98 (3)
I8—Ag6—Ag12	51.29 (3)	O26—Ag37—Ag38	84.6 (2)
O13—Ag6—Ag5	133.8 (3)	O19^iii^—Ag37—Ag38	121.6 (3)
O6—Ag6—Ag5	76.9 (3)	I3—Ag37—Ag38	57.84 (3)
O11—Ag6—Ag5	62.4 (2)	I6^iii^—Ag37—Ag38	138.84 (5)
I6^iii^—Ag6—Ag5	139.87 (4)	Ag7^iii^—Ag37—Ag38	104.70 (4)
I5—Ag6—Ag5	51.81 (3)	O27—Ag38—O32	79.0 (3)
I8—Ag6—Ag5	77.20 (3)	O27—Ag38—I3	126.8 (2)
Ag12—Ag6—Ag5	111.78 (4)	O32—Ag38—I3	141.8 (2)
O7—Ag7—O5^ii^	81.5 (4)	O27—Ag38—Ag37	73.7 (2)
O7—Ag7—I6	141.4 (3)	O32—Ag38—Ag37	146.3 (2)
O5^ii^—Ag7—I6	133.6 (3)	I3—Ag38—Ag37	54.21 (3)
O7—Ag7—Ag37^ii^	128.2 (3)	O27—Ag38—I1^ii^	136.8 (2)
O5^ii^—Ag7—Ag37^ii^	112.7 (3)	O32—Ag38—I1^ii^	75.7 (2)
I6—Ag7—Ag37^ii^	60.75 (3)	I3—Ag38—I1^ii^	93.83 (4)
O7—Ag7—Ag8	83.1 (3)	Ag37—Ag38—I1^ii^	138.02 (4)
O5^ii^—Ag7—Ag8	145.5 (3)	O27—Ag38—Ag3^iv^	132.8 (2)
I6—Ag7—Ag8	58.78 (3)	O32—Ag38—Ag3^iv^	56.6 (2)
Ag37^ii^—Ag7—Ag8	101.07 (4)	I3—Ag38—Ag3^iv^	88.16 (4)
O7—Ag7—I3^ii^	85.2 (3)	Ag37—Ag38—Ag3^iv^	137.71 (5)
O5^ii^—Ag7—I3^ii^	80.6 (3)	I1^ii^—Ag38—Ag3^iv^	50.48 (3)
I6—Ag7—I3^ii^	112.37 (5)	O27—Ag38—Ag39	51.6 (2)
Ag37^ii^—Ag7—I3^ii^	51.77 (3)	O32—Ag38—Ag39	70.3 (2)
Ag8—Ag7—I3^ii^	128.50 (5)	I3—Ag38—Ag39	146.88 (5)
O7—Ag7—Ag29	80.2 (2)	Ag37—Ag38—Ag39	105.78 (4)
O5^ii^—Ag7—Ag29	60.2 (3)	I1^ii^—Ag38—Ag39	86.75 (4)
I6—Ag7—Ag29	102.20 (4)	Ag3^iv^—Ag38—Ag39	116.48 (4)
Ag37^ii^—Ag7—Ag29	151.00 (5)	O31—Ag39—O24^ii^	82.0 (3)
Ag8—Ag7—Ag29	86.82 (4)	O31—Ag39—O27	99.3 (3)
I3^ii^—Ag7—Ag29	139.69 (4)	O24^ii^—Ag39—O27	122.3 (3)
O9—Ag8—O16	79.4 (3)	O31—Ag39—I5	132.5 (2)
O9—Ag8—I6	127.2 (3)	O24^ii^—Ag39—I5	138.8 (3)
O16—Ag8—I6	138.3 (2)	O27—Ag39—I5	79.4 (2)
O9—Ag8—Ag7	73.9 (3)	O31—Ag39—Ag34	157.0 (2)
O16—Ag8—Ag7	143.8 (2)	O24^ii^—Ag39—Ag34	78.1 (2)
I6—Ag8—Ag7	54.33 (3)	O27—Ag39—Ag34	81.9 (2)
O9—Ag8—Ag9^iv^	56.8 (3)	I5—Ag39—Ag34	70.52 (3)
O16—Ag8—Ag9^iv^	68.5 (2)	O31—Ag39—Ag38	79.3 (2)
I6—Ag8—Ag9^iv^	150.99 (5)	O24^ii^—Ag39—Ag38	154.6 (3)
Ag7—Ag8—Ag9^iv^	113.71 (4)	O27—Ag39—Ag38	45.6 (2)
O9—Ag8—I7	75.0 (3)	I5—Ag39—Ag38	66.08 (3)
O16—Ag8—I7	119.2 (2)	Ag34—Ag39—Ag38	115.42 (4)
I6—Ag8—I7	100.13 (4)	O29—Ag40—O31^iii^	85.5 (3)
Ag7—Ag8—I7	76.91 (4)	O29—Ag40—I2	137.4 (2)
Ag9^iv^—Ag8—I7	51.24 (3)	O31^iii^—Ag40—I2	128.2 (2)
O9—Ag8—I9^iv^	140.8 (3)	O29—Ag40—Ag40^ix^	128.2 (2)
O16—Ag8—I9^iv^	74.2 (2)	O31^iii^—Ag40—Ag40^ix^	119.5 (2)
I6—Ag8—I9^iv^	91.25 (4)	I2—Ag40—Ag40^ix^	62.40 (4)
Ag7—Ag8—I9^iv^	140.53 (5)	O29—Ag40—I2^ix^	86.3 (2)
Ag9^iv^—Ag8—I9^iv^	86.51 (4)	O31^iii^—Ag40—I2^ix^	85.3 (2)
I7—Ag8—I9^iv^	93.21 (4)	I2—Ag40—I2^ix^	117.65 (4)
O9—Ag8—Ag13	131.2 (3)	Ag40^ix^—Ag40—I2^ix^	55.24 (4)
O16—Ag8—Ag13	56.8 (2)	O29—Ag40—Ag22	71.3 (2)
I6—Ag8—Ag13	83.73 (4)	O31^iii^—Ag40—Ag22	72.7 (2)
Ag7—Ag8—Ag13	130.23 (5)	I2—Ag40—Ag22	92.77 (4)
Ag9^iv^—Ag8—Ag13	115.64 (4)	Ag40^ix^—Ag40—Ag22	155.05 (6)
I7—Ag8—Ag13	143.00 (4)	I2^ix^—Ag40—Ag22	149.47 (4)
I9^iv^—Ag8—Ag13	49.81 (3)	O1^vi^—C1—O2	122.8 (11)
O17—Ag9—O3^viii^	80.9 (3)	O1^vi^—C1—O3	121.0 (11)
O17—Ag9—I7^vi^	136.2 (3)	O2—C1—O3	116.1 (11)
O3^viii^—Ag9—I7^vi^	136.3 (2)	O4—C2—O6	121.2 (13)
O17—Ag9—Ag4^viii^	160.9 (3)	O4—C2—O5	119.7 (12)
O3^viii^—Ag9—Ag4^viii^	81.7 (2)	O6—C2—O5	119.1 (12)
I7^vi^—Ag9—Ag4^viii^	62.84 (3)	O9—C3—O7	122.6 (12)
O17—Ag9—Ag8^vi^	84.5 (3)	O9—C3—O8	118.7 (12)
O3^viii^—Ag9—Ag8^vi^	158.1 (2)	O7—C3—O8	118.7 (12)
I7^vi^—Ag9—Ag8^vi^	64.39 (3)	O12—C4—O10^i^	122.9 (12)
Ag4^viii^—Ag9—Ag8^vi^	109.69 (4)	O12—C4—O11	118.7 (12)
O10—Ag10—O17^vii^	89.4 (4)	O10^i^—C4—O11	118.4 (11)
O10—Ag10—I10	137.4 (2)	O15—C5—O14	121.2 (11)
O17^vii^—Ag10—I10	127.2 (2)	O15—C5—O13^ii^	120.8 (11)
O10—Ag10—Ag30^ix^	131.1 (2)	O14—C5—O13^ii^	117.7 (11)
O17^vii^—Ag10—Ag30^ix^	111.5 (3)	O17^iv^—C6—O16	123.4 (13)
I10—Ag10—Ag30^ix^	60.78 (4)	O17^iv^—C6—O18^iv^	119.7 (13)
O10—Ag10—I4^ix^	86.7 (2)	O16—C6—O18^iv^	116.7 (13)
O17^vii^—Ag10—I4^ix^	84.7 (2)	O20^iv^—C7—O21	122.0 (12)
I10—Ag10—I4^ix^	114.46 (4)	O20^iv^—C7—O19	121.1 (12)
Ag30^ix^—Ag10—I4^ix^	54.06 (3)	O21—C7—O19	116.9 (12)
O10—Ag10—Ag11^x^	81.1 (2)	O23—C8—O22	122.7 (12)
O17^vii^—Ag10—Ag11^x^	147.9 (3)	O23—C8—O24	121.1 (12)
I10—Ag10—Ag11^x^	56.37 (3)	O22—C8—O24	116.2 (12)
Ag30^ix^—Ag10—Ag11^x^	97.51 (4)	O27—C9—O26	123.0 (12)
I4^ix^—Ag10—Ag11^x^	124.80 (4)	O27—C9—O25	119.9 (12)
O10—Ag10—Ag32^vi^	75.4 (2)	O26—C9—O25	117.0 (11)
O17^vii^—Ag10—Ag32^vi^	67.4 (3)	O30—C10—O28^ix^	123.3 (12)
I10—Ag10—Ag32^vi^	97.49 (4)	O30—C10—O29	119.4 (12)
Ag30^ix^—Ag10—Ag32^vi^	153.06 (5)	O28^ix^—C10—O29	117.2 (11)
I4^ix^—Ag10—Ag32^vi^	146.54 (4)	O33^ii^—C11—O32	120.2 (12)
Ag11^x^—Ag10—Ag32^vi^	80.60 (4)	O33^ii^—C11—O31	118.9 (12)
O12—Ag11—O22	81.5 (3)	O32—C11—O31	120.9 (12)
O12—Ag11—I10^i^	126.1 (3)	O36—C12—O34	121.8 (13)
O22—Ag11—I10^i^	138.0 (2)	O36—C12—O35	118.8 (13)
O12—Ag11—Ag12	57.5 (3)	O34—C12—O35	119.4 (13)
O22—Ag11—Ag12	69.8 (2)	C1^iv^—O1—Ag22^iv^	121.6 (8)
I10^i^—Ag11—Ag12	149.72 (5)	C1^iv^—O1—Ag27^ii^	113.6 (8)
O12—Ag11—Ag10^i^	73.9 (2)	Ag22^iv^—O1—Ag27^ii^	118.9 (4)
O22—Ag11—Ag10^i^	146.6 (2)	C1—O2—Ag21	126.6 (8)
I10^i^—Ag11—Ag10^i^	53.41 (3)	C1—O2—Ag5^x^	93.9 (7)
Ag12—Ag11—Ag10^i^	112.89 (4)	Ag21—O2—Ag5^x^	136.9 (4)
O12—Ag11—I8	75.9 (2)	C1—O2—Ag25	115.2 (8)
O22—Ag11—I8	120.1 (2)	Ag21—O2—Ag25	74.9 (3)
I10^i^—Ag11—I8	98.81 (4)	Ag5^x^—O2—Ag25	74.6 (2)
Ag12—Ag11—I8	51.20 (3)	C1—O3—Ag9^vii^	121.8 (8)
Ag10^i^—Ag11—I8	75.62 (3)	C1—O3—Ag26^vi^	117.3 (8)
O12—Ag11—Ag15	132.2 (2)	Ag9^vii^—O3—Ag26^vi^	102.1 (4)
O22—Ag11—Ag15	55.4 (2)	C1—O3—Ag5^x^	95.5 (7)
I10^i^—Ag11—Ag15	84.82 (4)	Ag9^vii^—O3—Ag5^x^	120.9 (4)
Ag12—Ag11—Ag15	115.09 (4)	Ag26^vi^—O3—Ag5^x^	97.8 (3)
Ag10^i^—Ag11—Ag15	131.93 (4)	C2—O4—Ag1	130.4 (9)
I8—Ag11—Ag15	140.93 (4)	C2—O4—Ag21^i^	126.0 (9)
O23—Ag12—O13	114.7 (4)	Ag1—O4—Ag21^i^	92.3 (3)
O23—Ag12—I8	134.8 (3)	C2—O4—Ag2	90.9 (8)
O13—Ag12—I8	110.6 (3)	Ag1—O4—Ag2	85.9 (3)
O23—Ag12—Ag11	82.6 (2)	Ag21^i^—O4—Ag2	128.5 (4)
O13—Ag12—Ag11	135.3 (2)	C2—O5—Ag7^iii^	126.7 (9)
I8—Ag12—Ag11	65.74 (4)	C2—O5—Ag24^iii^	111.7 (9)
O23—Ag12—Ag6	162.5 (3)	Ag7^iii^—O5—Ag24^iii^	102.4 (4)
O13—Ag12—Ag6	47.8 (3)	C2—O5—Ag2	95.7 (8)
I8—Ag12—Ag6	62.72 (3)	Ag7^iii^—O5—Ag2	117.3 (4)
Ag11—Ag12—Ag6	109.78 (4)	Ag24^iii^—O5—Ag2	100.5 (4)
O15—Ag13—O25	74.4 (4)	C2—O6—Ag25^i^	118.7 (9)
O15—Ag13—I9^iv^	141.1 (3)	C2—O6—Ag29^iii^	107.0 (9)
O25—Ag13—I9^iv^	144.6 (2)	Ag25^i^—O6—Ag29^iii^	119.1 (4)
O15—Ag13—Ag33	61.7 (3)	C2—O6—Ag6	114.9 (8)
O25—Ag13—Ag33	68.0 (3)	Ag25^i^—O6—Ag6	101.2 (4)
I9^iv^—Ag13—Ag33	121.59 (5)	Ag29^iii^—O6—Ag6	93.0 (4)
O15—Ag13—I11^ii^	95.3 (3)	C3—O7—Ag7	123.3 (8)
O25—Ag13—I11^ii^	84.8 (2)	C3—O7—Ag3	112.6 (8)
I9^iv^—Ag13—I11^ii^	90.54 (4)	Ag7—O7—Ag3	114.4 (4)
Ag33—Ag13—I11^ii^	147.81 (4)	C3—O8—Ag23	122.1 (8)
O15—Ag13—Ag14^iv^	131.5 (3)	C3—O8—Ag27	110.0 (8)
O25—Ag13—Ag14^iv^	79.8 (2)	Ag23—O8—Ag27	113.0 (4)
I9^iv^—Ag13—Ag14^iv^	73.29 (4)	C3—O8—Ag4^v^	106.9 (8)
Ag33—Ag13—Ag14^iv^	70.68 (4)	Ag23—O8—Ag4^v^	102.9 (4)
I11^ii^—Ag13—Ag14^iv^	122.63 (4)	Ag27—O8—Ag4^v^	98.8 (3)
O15—Ag13—Ag8	77.3 (3)	C3—O9—Ag8	126.6 (9)
O25—Ag13—Ag8	149.8 (3)	C3—O9—Ag28	133.2 (9)
I9^iv^—Ag13—Ag8	64.36 (3)	Ag8—O9—Ag28	90.2 (3)
Ag33—Ag13—Ag8	89.43 (4)	C4^x^—O10—Ag10	126.1 (8)
I11^ii^—Ag13—Ag8	108.24 (4)	C4^x^—O10—Ag5^x^	111.3 (8)
Ag14^iv^—Ag13—Ag8	112.45 (4)	Ag10—O10—Ag5^x^	111.1 (4)
O18—Ag14—O19^xi^	87.5 (3)	C4^x^—O10—Ag26^vi^	109.1 (8)
O18—Ag14—O26^vi^	78.6 (3)	Ag10—O10—Ag26^vi^	97.1 (3)
O19^xi^—Ag14—O26^vi^	74.8 (3)	Ag5^x^—O10—Ag26^vi^	96.8 (3)
O18—Ag14—I14	157.5 (3)	C4—O11—Ag25^i^	126.0 (8)
O19^xi^—Ag14—I14	109.0 (2)	C4—O11—Ag30	105.8 (8)
O26^vi^—Ag14—I14	90.8 (2)	Ag25^i^—O11—Ag30	112.6 (4)
O18—Ag14—I10^viii^	104.7 (3)	C4—O11—Ag6	107.7 (8)
O19^xi^—Ag14—I10^viii^	85.0 (2)	Ag25^i^—O11—Ag6	99.2 (3)
O26^vi^—Ag14—I10^viii^	159.4 (2)	Ag30—O11—Ag6	103.0 (3)
I14—Ag14—I10^viii^	92.23 (4)	C4—O12—Ag11	127.9 (9)
O18—Ag14—Ag19	135.0 (2)	C4—O12—Ag31^i^	133.8 (8)
O19^xi^—Ag14—Ag19	48.0 (2)	Ag11—O12—Ag31^i^	88.9 (3)
O26^vi^—Ag14—Ag19	82.8 (2)	C5^iii^—O13—Ag30	125.2 (9)
I14—Ag14—Ag19	61.53 (3)	C5^iii^—O13—Ag6	122.5 (8)
I10^viii^—Ag14—Ag19	80.76 (4)	Ag30—O13—Ag6	108.3 (4)
O18—Ag14—Ag13^vi^	77.6 (3)	C5^iii^—O13—Ag12	110.9 (8)
O19^xi^—Ag14—Ag13^vi^	142.8 (2)	Ag30—O13—Ag12	92.2 (4)
O26^vi^—Ag14—Ag13^vi^	69.0 (2)	Ag6—O13—Ag12	84.1 (4)
I14—Ag14—Ag13^vi^	80.08 (4)	C5—O14—Ag29	113.3 (8)
I10^viii^—Ag14—Ag13^vi^	131.61 (4)	C5—O14—Ag28	128.4 (8)
Ag19—Ag14—Ag13^vi^	131.82 (4)	Ag29—O14—Ag28	96.0 (4)
O21^xii^—Ag15—O28^ix^	78.2 (3)	C5—O15—Ag13	129.8 (9)
O21^xii^—Ag15—I13	141.8 (2)	C5—O15—Ag34	120.1 (9)
O28^ix^—Ag15—I13	139.6 (2)	Ag13—O15—Ag34	109.4 (5)
O21^xii^—Ag15—Ag35^ix^	60.8 (3)	C6—O16—Ag8	130.2 (9)
O28^ix^—Ag15—Ag35^ix^	74.4 (2)	C6—O16—Ag33	95.6 (9)
I13—Ag15—Ag35^ix^	123.41 (4)	Ag8—O16—Ag33	130.1 (4)
O21^xii^—Ag15—I12^ii^	89.6 (3)	C6—O16—Ag28	127.2 (9)
O28^ix^—Ag15—I12^ii^	81.8 (2)	Ag8—O16—Ag28	87.6 (3)
I13—Ag15—I12^ii^	91.30 (4)	Ag33—O16—Ag28	75.8 (3)
Ag35^ix^—Ag15—I12^ii^	144.96 (4)	C6^vi^—O17—Ag9	117.4 (9)
O21^xii^—Ag15—Ag16	130.5 (2)	C6^vi^—O17—Ag10^viii^	114.8 (9)
O28^ix^—Ag15—Ag16	79.0 (2)	Ag9—O17—Ag10^viii^	121.9 (4)
I13—Ag15—Ag16	75.04 (3)	C6^vi^—O18—Ag32^xi^	118.7 (9)
Ag35^ix^—Ag15—Ag16	70.96 (3)	C6^vi^—O18—Ag14	116.9 (9)
I12^ii^—Ag15—Ag16	129.35 (4)	Ag32^xi^—O18—Ag14	107.9 (4)
O21^xii^—Ag15—Ag11	78.8 (2)	C6^vi^—O18—Ag33^vi^	94.4 (8)
O28^ix^—Ag15—Ag11	156.0 (2)	Ag32^xi^—O18—Ag33^vi^	119.2 (4)
I13—Ag15—Ag11	64.11 (3)	Ag14—O18—Ag33^vi^	97.7 (4)
Ag35^ix^—Ag15—Ag11	88.30 (4)	C7—O19—Ag19^xi^	105.2 (8)
I12^ii^—Ag15—Ag11	104.95 (4)	C7—O19—Ag37^ii^	130.9 (9)
Ag16—Ag15—Ag11	111.47 (4)	Ag19^xi^—O19—Ag37^ii^	97.9 (4)
O29—Ag16—O24	77.0 (3)	C7—O19—Ag14^xi^	123.9 (8)
O29—Ag16—I15^iii^	165.1 (2)	Ag19^xi^—O19—Ag14^xi^	80.9 (3)
O24—Ag16—I15^iii^	110.7 (2)	Ag37^ii^—O19—Ag14^xi^	101.9 (4)
O29—Ag16—Ag22	77.1 (2)	C7^vi^—O20—Ag32^vi^	115.0 (8)
O24—Ag16—Ag22	143.3 (2)	C7^vi^—O20—Ag31	129.7 (8)
I15^iii^—Ag16—Ag22	89.99 (4)	Ag32^vi^—O20—Ag31	93.9 (3)
O29—Ag16—I16	90.0 (2)	C7—O21—Ag36	121.8 (8)
O24—Ag16—I16	151.4 (3)	C7—O21—Ag15^xiv^	127.4 (8)
I15^iii^—Ag16—I16	88.21 (4)	Ag36—O21—Ag15^xiv^	110.2 (4)
Ag22—Ag16—I16	53.06 (3)	C8—O22—Ag11	129.8 (9)
O29—Ag16—Ag15	64.0 (2)	C8—O22—Ag35^ix^	95.9 (8)
O24—Ag16—Ag15	77.3 (2)	Ag11—O22—Ag35^ix^	132.0 (4)
I15^iii^—Ag16—Ag15	129.34 (4)	C8—O22—Ag31^i^	122.5 (8)
Ag22—Ag16—Ag15	113.22 (4)	Ag11—O22—Ag31^i^	88.7 (3)
I16—Ag16—Ag15	74.05 (3)	Ag35^ix^—O22—Ag31^i^	75.1 (3)
O25—Ag17—O23^ii^	85.0 (3)	C8—O23—Ag12	121.3 (9)
O25—Ag17—I15	136.3 (2)	C8—O23—Ag17^iii^	111.2 (8)
O23^ii^—Ag17—I15	128.8 (2)	Ag12—O23—Ag17^iii^	122.2 (4)
O25—Ag17—Ag27^ii^	129.8 (2)	C8—O23—Ag34^iii^	107.3 (9)
O23^ii^—Ag17—Ag27^ii^	119.1 (2)	Ag12—O23—Ag34^iii^	93.1 (3)
I15—Ag17—Ag27^ii^	62.90 (3)	Ag17^iii^—O23—Ag34^iii^	92.9 (3)
O25—Ag17—I11^ii^	88.6 (2)	C8—O24—Ag39^iii^	126.3 (8)
O23^ii^—Ag17—I11^ii^	84.6 (2)	C8—O24—Ag16	113.6 (8)
I15—Ag17—I11^ii^	117.69 (4)	Ag39^iii^—O24—Ag16	99.6 (4)
Ag27^ii^—Ag17—I11^ii^	54.79 (3)	C8—O24—Ag35^ix^	95.1 (8)
O34^iv^—Ag18—O32	78.3 (3)	Ag39^iii^—O24—Ag35^ix^	122.4 (4)
O34^iv^—Ag18—O27	80.2 (3)	Ag16—O24—Ag35^ix^	96.8 (3)
O32—Ag18—O27	72.9 (3)	C9—O25—Ag17	111.5 (8)
O34^iv^—Ag18—I15	165.0 (3)	C9—O25—Ag13	121.2 (8)
O32—Ag18—I15	87.6 (2)	Ag17—O25—Ag13	108.8 (4)
O27—Ag18—I15	100.7 (2)	C9—O25—Ag34	110.4 (8)
O34^iv^—Ag18—I16^iv^	81.7 (2)	Ag17—O25—Ag34	98.7 (3)
O32—Ag18—I16^iv^	103.8 (2)	Ag13—O25—Ag34	103.7 (4)
O27—Ag18—I16^iv^	161.8 (2)	C9—O26—Ag37	122.2 (8)
I15—Ag18—I16^iv^	96.91 (4)	C9—O26—Ag33	113.0 (8)
O34^iv^—Ag18—I14^iv^	94.9 (3)	Ag37—O26—Ag33	111.7 (4)
O32—Ag18—I14^iv^	155.4 (2)	C9—O26—Ag14^iv^	109.9 (8)
O27—Ag18—I14^iv^	82.7 (2)	Ag37—O26—Ag14^iv^	101.0 (3)
I15—Ag18—I14^iv^	100.05 (4)	Ag33—O26—Ag14^iv^	94.6 (3)
I16^iv^—Ag18—I14^iv^	98.54 (4)	C9—O27—Ag38	133.0 (9)
O34^iv^—Ag18—Ag23^iv^	82.8 (2)	C9—O27—Ag18	124.7 (8)
O32—Ag18—Ag23^iv^	51.3 (2)	Ag38—O27—Ag18	88.6 (3)
O27—Ag18—Ag23^iv^	123.8 (2)	C9—O27—Ag39	106.7 (8)
I15—Ag18—Ag23^iv^	84.41 (4)	Ag38—O27—Ag39	82.8 (3)
I16^iv^—Ag18—Ag23^iv^	53.68 (3)	Ag18—O27—Ag39	114.4 (4)
I14^iv^—Ag18—Ag23^iv^	152.21 (4)	C10^ix^—O28—Ag20^iii^	121.7 (8)
O34—Ag19—O19^xi^	114.5 (4)	C10^ix^—O28—Ag15^ix^	111.8 (8)
O34—Ag19—I12^x^	117.8 (3)	Ag20^iii^—O28—Ag15^ix^	111.6 (4)
O19^xi^—Ag19—I12^x^	121.3 (2)	C10—O29—Ag35^ix^	122.2 (8)
O34—Ag19—I14	97.5 (2)	C10—O29—Ag40	108.3 (8)
O19^xi^—Ag19—I14	104.5 (2)	Ag35^ix^—O29—Ag40	113.1 (4)
I12^x^—Ag19—I14	93.67 (4)	C10—O29—Ag16	109.1 (8)
O34—Ag19—Ag14	122.6 (3)	Ag35^ix^—O29—Ag16	103.1 (3)
O19^xi^—Ag19—Ag14	51.1 (2)	Ag40—O29—Ag16	97.9 (3)
I12^x^—Ag19—Ag14	113.16 (4)	C10—O30—Ag21	127.3 (9)
I14—Ag19—Ag14	53.82 (3)	C10—O30—Ag1^x^	130.3 (8)
O28^ii^—Ag20—O35^iii^	82.1 (4)	Ag21—O30—Ag1^x^	91.9 (3)
O28^ii^—Ag20—I12	146.7 (2)	C11—O31—Ag39	123.2 (8)
O35^iii^—Ag20—I12	117.4 (3)	C11—O31—Ag40^ii^	112.4 (8)
O28^ii^—Ag20—I12^xiii^	86.8 (2)	Ag39—O31—Ag40^ii^	120.2 (4)
O35^iii^—Ag20—I12^xiii^	93.5 (3)	C11—O32—Ag38	130.8 (9)
I12—Ag20—I12^xiii^	116.52 (4)	C11—O32—Ag23^iv^	93.9 (8)
O28^ii^—Ag20—Ag20^xiii^	134.3 (3)	Ag38—O32—Ag23^iv^	132.2 (4)
O35^iii^—Ag20—Ag20^xiii^	118.4 (3)	C11—O32—Ag18	122.9 (8)
I12—Ag20—Ag20^xiii^	62.66 (4)	Ag38—O32—Ag18	88.6 (3)
I12^xiii^—Ag20—Ag20^xiii^	53.86 (4)	Ag23^iv^—O32—Ag18	77.2 (3)
O28^ii^—Ag20—Ag21^i^	85.4 (2)	C11^iii^—O33—Ag22	127.7 (9)
O35^iii^—Ag20—Ag21^i^	127.6 (3)	C11^iii^—O33—Ag4	115.3 (9)
I12—Ag20—Ag21^i^	61.23 (3)	Ag22—O33—Ag4	99.5 (4)
I12^xiii^—Ag20—Ag21^i^	136.37 (5)	C11^iii^—O33—Ag23^v^	92.3 (8)
Ag20^xiii^—Ag20—Ag21^i^	106.51 (5)	Ag22—O33—Ag23^v^	121.9 (4)
O30—Ag21—O2	79.1 (3)	Ag4—O33—Ag23^v^	97.2 (3)
O30—Ag21—O4^x^	74.1 (3)	C12—O34—Ag19	110.2 (9)
O2—Ag21—O4^x^	74.3 (3)	C12—O34—Ag18^vi^	129.5 (9)
O30—Ag21—I12^x^	125.9 (2)	Ag19—O34—Ag18^vi^	97.1 (4)
O2—Ag21—I12^x^	136.8 (2)	C12—O35—Ag20^ii^	131.5 (9)
O4^x^—Ag21—I12^x^	79.9 (2)	C12—O35—Ag36^xi^	122.3 (9)
O30—Ag21—Ag20^x^	73.6 (2)	Ag20^ii^—O35—Ag36^xi^	103.4 (4)
O2—Ag21—Ag20^x^	143.1 (2)	C12—O35—Ag2^ii^	106.1 (9)
O4^x^—Ag21—Ag20^x^	74.6 (2)	Ag20^ii^—O35—Ag2^ii^	96.9 (4)
I12^x^—Ag21—Ag20^x^	53.94 (3)	Ag36^xi^—O35—Ag2^ii^	79.1 (4)
O30—Ag21—Ag25	127.2 (2)	C12—O36—Ag24	123.0 (9)
O2—Ag21—Ag25	54.2 (2)	C12—O36—Ag3	127.8 (9)
O4^x^—Ag21—Ag25	70.9 (2)	Ag24—O36—Ag3	109.0 (4)
I12^x^—Ag21—Ag25	84.99 (4)	Ag3^v^—I1—Ag2^iii^	104.24 (4)
Ag20^x^—Ag21—Ag25	130.08 (4)	Ag3^v^—I1—Ag36^v^	89.58 (4)
O30—Ag21—Ag22	55.6 (2)	Ag2^iii^—I1—Ag36^v^	64.56 (4)
O2—Ag21—Ag22	70.8 (2)	Ag3^v^—I1—Ag1^iii^	149.04 (4)
O4^x^—Ag21—Ag22	122.5 (2)	Ag2^iii^—I1—Ag1^iii^	69.62 (4)
I12^x^—Ag21—Ag22	151.34 (5)	Ag36^v^—I1—Ag1^iii^	112.72 (4)
Ag20^x^—Ag21—Ag22	111.13 (4)	Ag3^v^—I1—Ag38^iii^	65.28 (4)
Ag25—Ag21—Ag22	117.60 (4)	Ag2^iii^—I1—Ag38^iii^	76.39 (4)
O30—Ag21—I16	75.0 (2)	Ag36^v^—I1—Ag38^iii^	126.35 (4)
O2—Ag21—I16	123.0 (2)	Ag1^iii^—I1—Ag38^iii^	83.94 (4)
O4^x^—Ag21—I16	140.2 (2)	Ag40—I2—Ag4	78.75 (4)
I12^x^—Ag21—I16	98.92 (4)	Ag40—I2—Ag1^x^	77.99 (4)
Ag20^x^—Ag21—I16	73.11 (3)	Ag4—I2—Ag1^x^	117.64 (4)
Ag25—Ag21—I16	148.87 (4)	Ag40—I2—Ag40^ix^	62.35 (4)
Ag22—Ag21—I16	52.54 (3)	Ag4—I2—Ag40^ix^	114.93 (4)
O30—Ag21—I14	142.3 (2)	Ag1^x^—I2—Ag40^ix^	103.32 (4)
O2—Ag21—I14	76.0 (2)	Ag40—I2—Ag35	126.08 (4)
O4^x^—Ag21—I14	124.2 (2)	Ag4—I2—Ag35	86.42 (3)
I12^x^—Ag21—I14	91.35 (4)	Ag1^x^—I2—Ag35	150.55 (4)
Ag20^x^—Ag21—I14	139.39 (4)	Ag40^ix^—I2—Ag35	78.70 (3)
Ag25—Ag21—I14	53.40 (3)	Ag37—I3—Ag38	67.95 (4)
Ag22—Ag21—I14	89.28 (4)	Ag37—I3—Ag36^iii^	83.76 (4)
I16—Ag21—I14	95.52 (4)	Ag38—I3—Ag36^iii^	112.46 (4)
O1^vi^—Ag22—O33	82.5 (3)	Ag37—I3—Ag2	116.41 (4)
O1^vi^—Ag22—I16	135.6 (2)	Ag38—I3—Ag2	79.14 (4)
O33—Ag22—I16	135.6 (3)	Ag36^iii^—I3—Ag2	159.74 (4)
O1^vi^—Ag22—Ag16	159.3 (2)	Ag37—I3—Ag3^iii^	127.86 (4)
O33—Ag22—Ag16	78.8 (2)	Ag38—I3—Ag3^iii^	161.60 (4)
I16—Ag22—Ag16	65.13 (3)	Ag36^iii^—I3—Ag3^iii^	81.11 (4)
O1^vi^—Ag22—Ag21	82.9 (2)	Ag2—I3—Ag3^iii^	84.43 (4)
O33—Ag22—Ag21	159.8 (3)	Ag37—I3—Ag7^iii^	61.85 (3)
I16—Ag22—Ag21	63.87 (3)	Ag38—I3—Ag7^iii^	108.36 (4)
Ag16—Ag22—Ag21	112.64 (4)	Ag36^iii^—I3—Ag7^iii^	109.16 (4)
O1^vi^—Ag22—Ag40	96.1 (2)	Ag2—I3—Ag7^iii^	81.01 (3)
O33—Ag22—Ag40	64.5 (3)	Ag3^iii^—I3—Ag7^iii^	76.84 (3)
I16—Ag22—Ag40	118.73 (4)	Ag30—I4—Ag34^iii^	81.99 (4)
Ag16—Ag22—Ag40	67.66 (3)	Ag30—I4—Ag31^i^	74.28 (4)
Ag21—Ag22—Ag40	103.41 (4)	Ag34^iii^—I4—Ag31^i^	115.44 (4)
O8—Ag23—O32^vi^	109.4 (3)	Ag30—I4—Ag10^ix^	61.77 (3)
O8—Ag23—O33^v^	75.6 (3)	Ag34^iii^—I4—Ag10^ix^	114.25 (4)
O32^vi^—Ag23—O33^v^	52.0 (3)	Ag31^i^—I4—Ag10^ix^	105.30 (4)
O8—Ag23—I16	136.2 (2)	Ag30—I4—Ag5^iii^	130.53 (4)
O32^vi^—Ag23—I16	111.5 (2)	Ag34^iii^—I4—Ag5^iii^	91.62 (4)
O33^v^—Ag23—I16	144.5 (2)	Ag31^i^—I4—Ag5^iii^	147.28 (4)
O8—Ag23—Ag3	69.2 (2)	Ag10^ix^—I4—Ag5^iii^	77.21 (3)
O32^vi^—Ag23—Ag3	58.2 (2)	Ag5—I5—Ag39	104.44 (4)
O33^v^—Ag23—Ag3	78.6 (2)	Ag5—I5—Ag1	70.03 (4)
I16—Ag23—Ag3	122.38 (4)	Ag39—I5—Ag1	80.84 (4)
O8—Ag23—Ag18^vi^	160.8 (2)	Ag5—I5—Ag6	67.44 (3)
O32^vi^—Ag23—Ag18^vi^	51.5 (2)	Ag39—I5—Ag6	161.34 (4)
O33^v^—Ag23—Ag18^vi^	90.8 (2)	Ag1—I5—Ag6	110.23 (4)
I16—Ag23—Ag18^vi^	61.30 (3)	Ag7—I6—Ag8	66.89 (4)
Ag3—Ag23—Ag18^vi^	95.20 (4)	Ag7—I6—Ag37^ii^	65.28 (4)
O8—Ag23—I15^iii^	82.2 (2)	Ag8—I6—Ag37^ii^	109.53 (4)
O32^vi^—Ag23—I15^iii^	123.7 (2)	Ag7—I6—Ag6^ii^	72.96 (4)
O33^v^—Ag23—I15^iii^	81.2 (2)	Ag8—I6—Ag6^ii^	112.38 (4)
I16—Ag23—I15^iii^	87.77 (4)	Ag37^ii^—I6—Ag6^ii^	99.26 (4)
Ag3—Ag23—I15^iii^	148.22 (4)	Ag7—I6—Ag32	123.20 (4)
Ag18^vi^—Ag23—I15^iii^	109.47 (4)	Ag8—I6—Ag32	80.53 (4)
O8—Ag23—Ag24	78.6 (2)	Ag37^ii^—I6—Ag32	85.53 (4)
O32^vi^—Ag23—Ag24	119.7 (2)	Ag6^ii^—I6—Ag32	163.28 (4)
O33^v^—Ag23—Ag24	146.2 (2)	Ag7—I6—Ag33^ii^	131.29 (4)
I16—Ag23—Ag24	68.21 (3)	Ag8—I6—Ag33^ii^	160.12 (4)
Ag3—Ag23—Ag24	71.92 (3)	Ag37^ii^—I6—Ag33^ii^	77.48 (3)
Ag18^vi^—Ag23—Ag24	107.84 (4)	Ag6^ii^—I6—Ag33^ii^	83.85 (3)
I15^iii^—Ag23—Ag24	116.54 (4)	Ag32—I6—Ag33^ii^	81.56 (3)
O36—Ag24—O5^ii^	88.3 (4)	Ag9^iv^—I7—Ag35^v^	108.57 (4)
O36—Ag24—I13	141.2 (3)	Ag9^iv^—I7—Ag31^iv^	80.64 (4)
O5^ii^—Ag24—I13	121.8 (3)	Ag35^v^—I7—Ag31^iv^	64.19 (3)
O36—Ag24—Ag29	148.0 (3)	Ag9^iv^—I7—Ag4^v^	63.67 (3)
O5^ii^—Ag24—Ag29	64.4 (3)	Ag35^v^—I7—Ag4^v^	89.99 (4)
I13—Ag24—Ag29	58.04 (3)	Ag31^iv^—I7—Ag4^v^	126.55 (4)
O36—Ag24—I12^ii^	118.6 (3)	Ag9^iv^—I7—Ag8	64.37 (3)
O5^ii^—Ag24—I12^ii^	89.4 (2)	Ag35^v^—I7—Ag8	149.45 (4)
I13—Ag24—I12^ii^	88.12 (4)	Ag31^iv^—I7—Ag8	85.27 (4)
Ag29—Ag24—I12^ii^	79.51 (4)	Ag4^v^—I7—Ag8	110.26 (4)
O36—Ag24—Ag23	57.8 (3)	Ag12—I8—Ag33	110.71 (4)
O5^ii^—Ag24—Ag23	133.5 (2)	Ag12—I8—Ag28	82.39 (4)
I13—Ag24—Ag23	83.41 (4)	Ag33—I8—Ag28	65.73 (3)
Ag29—Ag24—Ag23	130.45 (4)	Ag12—I8—Ag6	65.99 (4)
I12^ii^—Ag24—Ag23	133.17 (4)	Ag33—I8—Ag6	89.96 (4)
O11^x^—Ag25—O6^x^	79.4 (3)	Ag28—I8—Ag6	130.27 (4)
O11^x^—Ag25—O2	119.2 (3)	Ag12—I8—Ag11	63.06 (3)
O6^x^—Ag25—O2	99.0 (3)	Ag33—I8—Ag11	151.23 (4)
O11^x^—Ag25—I14	134.1 (2)	Ag28—I8—Ag11	85.50 (4)
O6^x^—Ag25—I14	141.5 (3)	Ag6—I8—Ag11	110.18 (4)
O2—Ag25—I14	81.2 (2)	Ag13^vi^—I9—Ag32^vi^	103.36 (4)
O11^x^—Ag25—Ag21	160.3 (2)	Ag13^vi^—I9—Ag26^vi^	87.69 (4)
O6^x^—Ag25—Ag21	85.3 (2)	Ag32^vi^—I9—Ag26^vi^	62.62 (3)
O2—Ag25—Ag21	51.0 (2)	Ag13^vi^—I9—Ag31	147.96 (4)
I14—Ag25—Ag21	64.85 (3)	Ag32^vi^—I9—Ag31	70.35 (3)
O11^x^—Ag25—Ag5^x^	67.4 (2)	Ag26^vi^—I9—Ag31	114.28 (4)
O6^x^—Ag25—Ag5^x^	82.1 (3)	Ag13^vi^—I9—Ag8^vi^	65.83 (4)
O2—Ag25—Ag5^x^	52.5 (2)	Ag32^vi^—I9—Ag8^vi^	78.44 (4)
I14—Ag25—Ag5^x^	123.97 (4)	Ag26^vi^—I9—Ag8^vi^	126.18 (4)
Ag21—Ag25—Ag5^x^	98.37 (4)	Ag31—I9—Ag8^vi^	82.18 (3)
O3^iv^—Ag26—O10^iv^	79.4 (3)	Ag10—I10—Ag11^x^	70.22 (4)
O3^iv^—Ag26—I11^ii^	106.6 (2)	Ag10—I10—Ag30^ix^	64.37 (4)
O10^iv^—Ag26—I11^ii^	162.0 (2)	Ag11^x^—I10—Ag30^ix^	108.77 (4)
O3^iv^—Ag26—I9^iv^	153.3 (2)	Ag10—I10—Ag29^x^	124.39 (4)
O10^iv^—Ag26—I9^iv^	88.8 (2)	Ag11^x^—I10—Ag29^x^	83.63 (4)
I11^ii^—Ag26—I9^iv^	91.93 (4)	Ag30^ix^—I10—Ag29^x^	80.35 (4)
O3^iv^—Ag26—Ag32	141.6 (2)	Ag10—I10—Ag14^vii^	79.57 (4)
O10^iv^—Ag26—Ag32	80.3 (2)	Ag11^x^—I10—Ag14^vii^	115.52 (4)
I11^ii^—Ag26—Ag32	85.04 (4)	Ag30^ix^—I10—Ag14^vii^	106.73 (4)
I9^iv^—Ag26—Ag32	57.17 (3)	Ag29^x^—I10—Ag14^vii^	154.60 (4)
O8—Ag27—O1^iii^	85.1 (3)	Ag27—I11—Ag28	79.43 (4)
O8—Ag27—I11	137.3 (2)	Ag27—I11—Ag26^iii^	80.72 (4)
O1^iii^—Ag27—I11	127.0 (2)	Ag28—I11—Ag26^iii^	119.27 (4)
O8—Ag27—Ag17^iii^	128.9 (2)	Ag27—I11—Ag17^iii^	61.83 (3)
O1^iii^—Ag27—Ag17^iii^	120.2 (2)	Ag28—I11—Ag17^iii^	105.19 (4)
I11—Ag27—Ag17^iii^	63.38 (3)	Ag26^iii^—I11—Ag17^iii^	114.20 (4)
O8—Ag27—I15^iii^	86.6 (2)	Ag27—I11—Ag13^iii^	122.19 (4)
O1^iii^—Ag27—I15^iii^	86.8 (2)	Ag28—I11—Ag13^iii^	153.98 (4)
I11—Ag27—I15^iii^	118.44 (4)	Ag26^iii^—I11—Ag13^iii^	81.20 (3)
Ag17^iii^—Ag27—I15^iii^	55.06 (3)	Ag17^iii^—I11—Ag13^iii^	77.16 (3)
O14—Ag28—O9	77.4 (4)	Ag20—I12—Ag19^i^	120.04 (4)
O14—Ag28—O16	80.8 (3)	Ag20—I12—Ag21^i^	64.83 (3)
O9—Ag28—O16	74.4 (3)	Ag19^i^—I12—Ag21^i^	82.59 (4)
O14—Ag28—I11	166.7 (3)	Ag20—I12—Ag20^xiii^	63.48 (4)
O9—Ag28—I11	98.4 (3)	Ag19^i^—I12—Ag20^xiii^	84.97 (4)
O16—Ag28—I11	85.9 (2)	Ag21^i^—I12—Ag20^xiii^	109.61 (4)
O14—Ag28—I8	85.5 (3)	Ag20—I12—Ag15^iii^	130.11 (4)
O9—Ag28—I8	162.8 (3)	Ag19^i^—I12—Ag15^iii^	84.83 (4)
O16—Ag28—I8	105.2 (2)	Ag21^i^—I12—Ag15^iii^	164.41 (4)
I11—Ag28—I8	98.73 (4)	Ag20^xiii^—I12—Ag15^iii^	78.30 (3)
O14—Ag28—Ag33	85.1 (3)	Ag20—I12—Ag24^iii^	81.27 (4)
O9—Ag28—Ag33	124.9 (2)	Ag19^i^—I12—Ag24^iii^	158.30 (4)
O16—Ag28—Ag33	51.2 (2)	Ag21^i^—I12—Ag24^iii^	112.73 (4)
I11—Ag28—Ag33	87.18 (4)	Ag20^xiii^—I12—Ag24^iii^	102.95 (4)
I8—Ag28—Ag33	54.47 (3)	Ag15^iii^—I12—Ag24^iii^	77.23 (3)
O14—Ag28—I13	91.9 (3)	Ag15—I13—Ag24	90.20 (4)
O9—Ag28—I13	78.4 (2)	Ag15—I13—Ag29	105.36 (4)
O16—Ag28—I13	152.8 (2)	Ag24—I13—Ag29	65.28 (4)
I11—Ag28—I13	99.65 (4)	Ag15—I13—Ag28	148.85 (4)
I8—Ag28—I13	100.34 (4)	Ag24—I13—Ag28	113.66 (4)
Ag33—Ag28—I13	154.77 (4)	Ag29—I13—Ag28	70.21 (3)
O14—Ag29—O6^ii^	119.4 (4)	Ag25—I14—Ag14	92.63 (4)
O14—Ag29—I13	101.0 (3)	Ag25—I14—Ag19	101.58 (4)
O6^ii^—Ag29—I13	136.3 (3)	Ag14—I14—Ag19	64.65 (3)
O14—Ag29—I10^i^	117.1 (3)	Ag25—I14—Ag18^vi^	144.30 (4)
O6^ii^—Ag29—I10^i^	83.1 (2)	Ag14—I14—Ag18^vi^	112.82 (4)
I13—Ag29—I10^i^	93.27 (4)	Ag19—I14—Ag18^vi^	70.24 (3)
O14—Ag29—Ag24	123.4 (3)	Ag25—I14—Ag21	61.75 (3)
O6^ii^—Ag29—Ag24	85.7 (2)	Ag14—I14—Ag21	128.69 (4)
I13—Ag29—Ag24	56.68 (3)	Ag19—I14—Ag21	77.33 (3)
I10^i^—Ag29—Ag24	115.57 (4)	Ag18^vi^—I14—Ag21	82.60 (3)
O14—Ag29—Ag7	70.3 (3)	Ag17—I15—Ag16^ii^	78.40 (4)
O6^ii^—Ag29—Ag7	74.4 (2)	Ag17—I15—Ag18	78.39 (4)
I13—Ag29—Ag7	107.45 (4)	Ag16^ii^—I15—Ag18	115.08 (4)
I10^i^—Ag29—Ag7	156.56 (5)	Ag17—I15—Ag27^ii^	62.03 (3)
Ag24—Ag29—Ag7	69.80 (4)	Ag16^ii^—I15—Ag27^ii^	114.94 (4)
O13—Ag30—O11	76.6 (3)	Ag18—I15—Ag27^ii^	105.28 (4)
O13—Ag30—I4	114.9 (3)	Ag17—I15—Ag23^ii^	126.19 (4)
O11—Ag30—I4	144.5 (2)	Ag16^ii^—I15—Ag23^ii^	88.27 (4)
O13—Ag30—I10^ix^	102.7 (3)	Ag18—I15—Ag23^ii^	150.50 (4)
O11—Ag30—I10^ix^	88.6 (2)	Ag27^ii^—I15—Ag23^ii^	78.17 (3)
I4—Ag30—I10^ix^	118.60 (5)	Ag22—I16—Ag23	108.58 (4)
O13—Ag30—Ag10^ix^	134.2 (3)	Ag22—I16—Ag18^vi^	82.22 (4)
O11—Ag30—Ag10^ix^	133.0 (2)	Ag23—I16—Ag18^vi^	65.03 (3)
I4—Ag30—Ag10^ix^	64.17 (3)	Ag22—I16—Ag21	63.58 (3)
I10^ix^—Ag30—Ag10^ix^	54.85 (3)	Ag23—I16—Ag21	148.35 (4)
O20—Ag31—O12^x^	77.9 (3)	Ag18^vi^—I16—Ag21	83.34 (4)
O20—Ag31—O22^x^	78.3 (3)	Ag22—I16—Ag16	61.81 (3)
O12^x^—Ag31—O22^x^	75.6 (3)	Ag23—I16—Ag16	89.39 (4)
O20—Ag31—I4^x^	165.3 (2)	Ag18^vi^—I16—Ag16	126.65 (4)
O12^x^—Ag31—I4^x^	101.3 (2)	Ag21—I16—Ag16	110.02 (4)
O22^x^—Ag31—I4^x^	87.2 (2)		

Symmetry codes: (i) −*x*, *y*−1/2, −*z*+1/2; (ii) *x*, −*y*+1/2, *z*−1/2; (iii) *x*, −*y*+1/2, *z*+1/2; (iv) −*x*+1, *y*−1/2, −*z*+1/2; (v) −*x*+1, −*y*+1, −*z*+1; (vi) −*x*+1, *y*+1/2, −*z*+1/2; (vii) *x*, −*y*+3/2, *z*+1/2; (viii) *x*, −*y*+3/2, *z*−1/2; (ix) −*x*, −*y*+1, −*z*+1; (x) −*x*, *y*+1/2, −*z*+1/2; (xi) −*x*+1, −*y*+1, −*z*; (xii) *x*−1, *y*, *z*; (xiii) −*x*, −*y*, −*z*+1; (xiv) *x*+1, *y*, *z*.
