# Supplementary material for: Crystal structure of silver carbonate iodide Ag10(CO3)3I4
Source: Acta Crystallogr E Crystallogr Commun. 2021 Jun 25;77(Pt 7):734–8. doi: 10.1107/S2056989021006022 (PMC8382059; doi:10.1107/S2056989021006022)
Supplement: Supplementary file 4 [file e-77-00734-sup3.pdf]

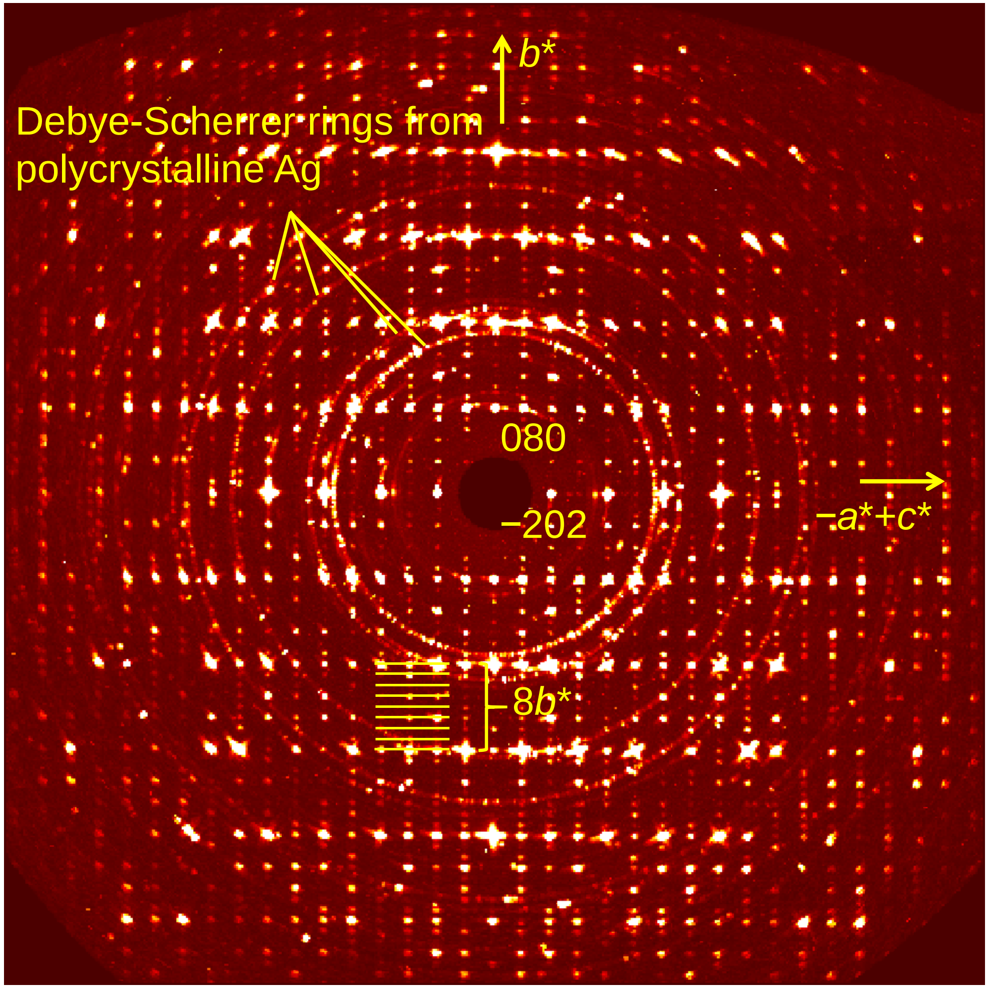

Figure S1
*b**- −*a**+*c** reciprocal lattice plane reconstructed from the oscillation XRD images, showing fundamental reflections every eight spots and the Debye-Scherrer rings from polycrystalline Ag.

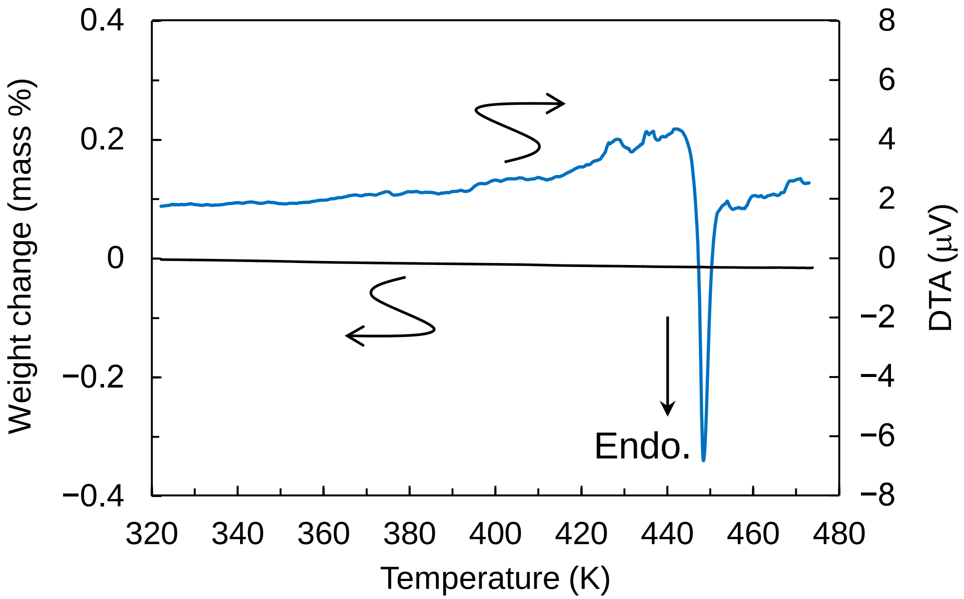

Figure S2
Thermogravimetric and differential thermal analysis curves of the starting mixture with a molar ratio of AgI:Ag_2CO_3 = 4:3 under constant flow of synthetic air.

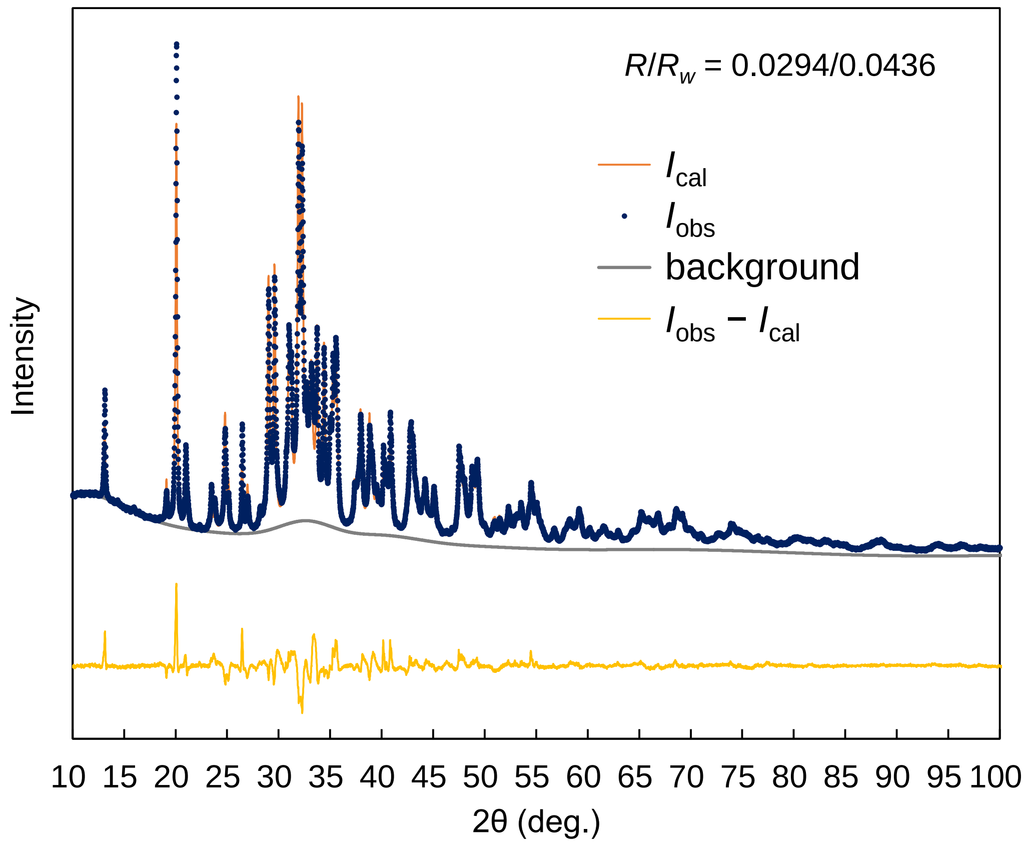

Figure S3

Fitting result of Rietveld analysis with the positional parameters fixed on the valued refined in the single-crystal XRD analysis at 90 K.
